# Inequalities in reproductive health care use in five West-African countries: A decomposition analysis of the wealth-based gaps

**DOI:** 10.1186/s12939-020-01167-7

**Published:** 2020-03-27

**Authors:** Oluwasegun Jko Ogundele, Milena Pavlova, Wim Groot

**Affiliations:** 1 Department of Health Services Research; CAPHRI, Maastricht University Medical Center; Faculty of Health, Medicine and Life Sciences; Maastricht University, PO Box 616, 6200MD, Maastricht, the Netherlands; 2 United Nations University-Maastricht Economic and Social Research Institute on Innovation and Technology, Maastricht, The Netherlands

**Keywords:** West Africa, Inequalities, Family Planning, Maternal Care

## Abstract

**Background:**

Family planning and maternal care services have become increasingly available in West Africa but the level of non-use remains high. This unfavorable outcome may be partly due to the unaffordability of reproductive health care services.

**Methods:**

Using the Demographic Health Survey data from Burkina Faso, Niger, Nigeria, Ghana, and Senegal, we perform a decomposition analysis to quantify the contribution of socio-demographic characteristics to disparities in exposure to mass media information on family planning, use of modern contraceptives, adequate antenatal care visits, facility-based childbirth and C-section between low-wealth and high-wealth women.

**Results:**

Our study shows that differences in maternal characteristics between the wealth groups explain at least 40% of the gap in exposure to mass media family planning information, 30% in modern contraceptive use, 24% of adequate antenatal care visits, 47% of the difference in facility-based childbirths, and 62% in C-section. Lack of information on pregnancy complications, living in rural residence, religion, lack of autonomy in health facility seeking decision, need to pay, and distance explains the disparity in reproductive health care use across all countries. In countries with complete fee exemption policies for specific groups in the population, Ghana, Niger, and Senegal, the inequality gaps between wealth groups in having an adequate number of antenatal care visits and facility-based childbirth are smaller than in countries with partial or no exemption policies. But this is not the case for C-section.

**Conclusions:**

There is evidence that current policies addressing the cost of maternal care services may increase the wealth-based inequality in maternal care use if socio-demographic differences are not addressed. Public health interventions are needed to target socio-demographic disparities and health facility seeking problems that disadvantage women in poor households.

## Statement of Significance

Issue

The level of non-use of reproductive health care services remains high in West Africa and the richer women use more reproductive health care services than the poor.

What is already known

High levels of inequity exist within countries, in family planning, modern contraceptives, antenatal care, facility-based deliveries and Caesarean section use by wealth quintile. National coverage statistics conceal extensive disparities in health services use between social groups.

What this Paper Adds

Evidence of the contribution of observed individual characteristics to the differences in reproductive health care services use between the poorest and richer women. Evidence that the countries with user fee exemptions need to target the vulnerable groups.

## Background

Many low- and middle-income countries have made efforts to foster equal access to health care. These efforts have not attained similar results [1]. In 2015, the World Health Organization and UNICEF reported differences in maternal health outcomes between low- and middle-income countries, with maternal deaths ranging between 599 and 849 per 100,000 live births in West and Central Africa combined. Tsui Amy, Brown [2] also provide evidence of the differences in the use of modern contraceptives within and across sub-Saharan African countries. For example, the prevalence of modern contraceptives is 18.5% in Kaduna, Nigeria and 26.4% in Lagos, Nigeria. The observed disparities in reproductive health outcomes and utilization are partly attributable to wealth. Evidence shows that the most vulnerable - the poor - are at a disadvantage, which prevents equal access to care [3–5].

There is evidence to suggest that the poor have peculiar characteristics, e.g. illiteracy, high parity and inefficient and insufficient exposure to the reproductive health information on mass media, which stymie their use of health care services in general [4, 6]. This applies to the use of reproductive health care services as well. While several studies have shown that better-off women have easier access to reproductive health care [5, 7–9], other studies have demonstrated that socio-demographic factors such as marital status, education, occupation, residence typology, attitude of health workers, and distance to health facility also contribute to the inequality in the use reproductive health care among women [9–16]. In addition, it is unclear to what extent these determinants explain the use of reproductive health care and contribute to the differences between the groups [6, 17]. In view of this evidence, a policy that aims to reduce inequality and helps to attain universal coverage of reproductive health care services should target the poorest people most in need of healthcare but should also take into account other determinants than wealth.

This study focuses on the gaps in the use of reproductive health care in five countries in West Africa, namely Burkina Faso, Ghana, Niger, Nigeria, and Senegal. These countries have a relatively high maternal mortality rate – in the range of 315-814 per 100 000 live births [1]. In addition, the countries have similar policies, either in place or in a process of implementation, to address reproductive health care services as part of the efforts to reduce maternal mortality [18, 19]. Burkina Faso is a low-income country with an estimated 18.6 million people in 2016 [20]. The gross national income per capita in 2013 was US$1,560. The maternal mortality ratio was estimated at 371 per 100,000 live births in 2015. Burkina Faso has a national maternal health care subsidy policy [21, 22]. The general government expenditure on health as a percentage of total government expenditure in 2014 was 11.2% and private expenditure on health was 47.7% of total expenditure on health [23].

Niger is a low-income country with an estimated population of 20.6 million and a gross national income per capita of $910 in 2013 [24]. The estimated maternal mortality ratio in Niger in 2010 was 590 per 100,000 live births. Niger implemented a user fees abolition policy for antenatal care, Caesarean sections and family planning as early as 2007 [25]. Niger’s general government expenditure on health as a percentage of total government expenditure was 11.1% and private expenditure on health as a percentage of total expenditure on health was 44.9% in 2011 [23].

Nigeria is classified as a lower-middle-income country. It has an estimated population of 186 million in 2016 and gross national income per capita in 2013 was US$5,360 [26]. The maternal mortality ratio in Nigeria was 814 per 100,000 live births in 2015 [26]. Nigeria has user fees but there are intermittent partially free maternal health care programs and a countrywide midwives scheme to improve the use of maternal health services [22, 27, 28]. The total expenditure on health per capita in 2014 was US$ 216.87. General government expenditure on health as a percentage of total government expenditure was 8.2% and the private expenditure on health as a percentage of total expenditure on health was 74.9% [23].

Ghana is a lower-middle-income country with a population estimate of 28.2 million in 2016 and a gross national income per capita of US$3,880 in 2013 [29]. The maternal mortality ratio in Ghana was 319 per 100,000 live births in 2015. In Ghana, antenatal care in all public health facilities is for free since 1998. Aa free delivery care policy initially in four regions (northern, upper east, upper west and central) was extended to all regions in 2005 [30]. The government of Ghana’s general expenditure on health as a percentage of total government expenditure was 6.8% in 2014 while private expenditure on health as a percentage of total expenditure on health accounted for 40.2% [23].

Senegal is another lower-middle-income country with an estimated population of 15.4 million in 2016, and gross national income per capita US$ 2,240 in 2013 [31]. The maternal mortality ratio was approximately 315 per 100,000 live births in 2015. Senegal has the free childbirth care and C-section policy [32]. The general government expenditure on health as a percentage of total government expenditure was 8.0% and private expenditure on health as a percentage of total expenditure on health accounted for 48.2% in 2014 [23].

This study investigates the wealth-based inequalities in the use of reproductive health care services among women in the selected countries and identifies factors that contribute to these inequalities. In particular, the probability of the use of reproductive health care services is decomposed for low-wealth and high-wealth women within the countries, and the relative disadvantage of different factors for the low-wealth group is analyzed. Socio-demographic, health and wealth contributing factors are included. We provide new knowledge by identifying characteristics that widen or narrow the inequality gap in reproductive health use. The study contributes to our understanding of the vulnerable groups that could be targeted in programs to “close the gap” between rich and poor. We also add to the current literature by presenting a cross-country comparison based on nationally representative datasets. The results may also be of interest to other low-middle income countries where reproductive health care use is on the policy agenda.

## Methods

Data from the Demographic Health Surveys (DHS) were used. DHS are cross-sectional, comparable and representative population-based surveys that gather varied information on reproductive health, nutrition and other demographics of respondents from low-middle income countries [33]. We used the latest DHS surveys available for Burkina Faso (2010), Niger (2012), Nigeria (2013), Ghana (2014) and Senegal (2016 DHS-VII).

All five DHS included in this study are household-based samples. The samples were selected in a similar manner. In particular, the DHS adopted a stratified two-stage cluster design and samples selected for enumeration were ensured to be representative of the countries. The households involved were drawn from a simplified list of households [34–36]. We only used data provided by women of reproductive age (15-49 years) who had given birth during the last 5 years before the survey and who were able to provide information on the use of reproductive health care services.

### Variables

The primary outcome variables of interest are binary indicators of whether the woman was exposed to family planning information via mass media, used modern contraceptives, made four or more antenatal care visits when pregnant, had facility-based childbirth, and had C-section childbirth. All five variables were dichotomized as 1 when a woman answered “Yes” to the respective question, and otherwise as 0 “No”.

We used the wealth index constructed by the DHS. This index is based on available information about household asset ownership, and housing and environmental conditions to analyze inequalities in reproductive health care services [37]. We dichotomized the wealth quintile index where low wealth represents the lowest quintile and high wealth represents women in the higher four wealth quintiles.

The socio-demographic covariates included were selected based on findings from previous empirical research on inequality and access to reproductive health care services [5, 8, 10, 13, 15, 16]. For independent variable with more than 5% missing cases, we added a separate dummy variable indicating a missing value in the analysis.

### Statistical analysis

To determine the extent to which wealth-based differences in exposure to mass media family planning information, use of modern contraceptives, adequate use of antenatal care visits, use of facility-based childbirth services and C-sections are due to differences in the observed respondents’ characteristics, we used a non-linear decomposition technique suggested by Fairlie [38]. The Fairlie decomposition technique is an extension of the Blinder-Oaxaca decomposition technique to binary outcome measures [38, 39]. Fairlie decomposition has been used to study group differences in a binary outcome variable including group differences in health facility utilization, racial differences in health outcome, rural-urban inequality, the disparity in health care utilization and insurance [6, 40, 41]. Fairlie decomposition generates simulated samples of data that pair observations (a one-to-one matching) from each wealth group and estimates the predicted differences between those samples [38]. The average contribution of each variable to the estimated gap from all iterated samples is reported as output [39].

Using the *fairlie* command in STATA 15, we draw a random subsample of the high-wealth group equal to the size of the low-wealth group with randomly ordered variables and 1,000 replications to decompose the explained part and to show the contribution of each of the variables to the gap. Using the high-wealth group subsample as the reference category, we report the results of the contribution of maternal characteristics to the gap in the outcome of reproductive health care services observed. An additional file shows the Logit results (See Supplementary 1, Additional file 1). We performed a sensitivity analysis by using the coefficients of the upper three quintiles against the lower two quintiles. The results are generally similar across specifications (See Supplementary Table 2, Additional File 1). The Fairlie decomposition method is detailed in (See Supplementary B, Additional File 1).

## Results

Table 1 shows the sample characteristics of respondents included in the analyses for each country. Of particular note is the proportion in the low-wealth group that has no education in Burkina Faso, Niger and Senegal, accounting for more than 80% of the respondents. More women in the high-wealth group reside in urban residences in Nigeria, Ghana, Senegal (at least 45%) compared with Burkina Faso and Niger (at most 23%). Also, more women live in rural locations in Niger and Burkina Faso compared with Ghana, Senegal, and Nigeria. On average, women are unlikely to have information on pregnancy complications in Burkina Faso, Niger, and Senegal. On average, women who needed permission to visit health facilities or who reported problems with money to pay, were more likely to be in Burkina Faso and Niger compared with Nigeria and Ghana. Although the problem of money to pay among women is comparable among women in the low-wealth group in Nigeria and Ghana. Generally, women are less likely to report having a problem to visit a health facility alone across all countries. Although descriptive, the statistics in Table 1 highlight some significant differences between women in different countries which may have an effect on use of reproductive health care services. See Supplementary Table 3, Additional file 1 for regional covariates included in the analysis. In the analysis, we included sampling weights to ensure population-representative analysis.

**Table 1:** Characteristics of respondents included in the analyses per country (weighted)

	Burkina Faso				Niger				Nigeria				Ghana				Senegal			
*Variable*	low-wealth		high-wealth		low-wealth		high-wealth		low-wealth		high-wealth		low-wealth		high-wealth		low-wealth		high-wealth	
	mean	sd	mean	sd	mean	sd	mean	sd	mean	sd	mean	sd	mean	sd	mean	sd	mean	sd	mean	sd
Age	30	8	29	7	29	7	29	7	29	8	30	7	31	8	31	7	29	8	30	7
Children ever born	4.74	2.64	3.82	2.39	4.94	2.78	4.70	2.84	4.79	2.93	3.87	2.52	4.14	2.42	3.08	1.94	4.47	2.72	3.43	2.22
No education	0.95	0.22	0.79	0.40	0.93	0.26	0.83	0.38	0.89	0.32	0.36	0.48	0.63	0.48	0.16	0.37	0.85	0.36	0.57	0.50
1^0^ education	0.05	0.21	0.13	0.34	0.06	0.24	0.11	0.32	0.09	0.29	0.22	0.42	0.21	0.41	0.19	0.39	0.11	0.32	0.25	0.44
Sec / higher education (ref.)	0.00	0.06	0.08	0.26	0.01	0.10	0.06	0.24	0.02	0.15	0.42	0.49	0.16	0.37	0.64	0.48	0.04	0.19	0.18	0.38
Urban (ref.)	0.02	0.13	0.23	0.42	0.00	0.06	0.18	0.38	0.05	0.22	0.45	0.50	0.10	0.30	0.56	0.50	0.03	0.18	0.49	0.50
Rural	0.98	0.13	0.77	0.42	1.00	0.06	0.82	0.38	0.95	0.22	0.55	0.50	0.90	0.30	0.44	0.50	0.97	0.18	0.51	0.50
Christian/Protestant/Catholic (ref.)	0.27	0.44	0.27	0.45	-	-	-	-	0.08	0.28	0.47	0.50	0.55	0.50	0.82	0.38	0.04	0.20	0.04	0.19
Islam	0.54	0.50	0.66	0.47	-	-	-	-	0.90	0.30	0.52	0.50	0.28	0.45	0.14	0.35	0.96	0.20	0.96	0.19
Traditional	0.17	0.37	0.06	0.24	-	-	-	-	0.01	0.12	0.01	0.10	0.17	0.38	0.04	0.19	-	-	-	-
Animist	-	-	-	-	-	-	-	-	-	-	-	-	-	-	-	-	0.02	0.12	0.00	0.05
No partner	0.03	0.17	0.03	0.18	0.02	0.15	0.02	0.16	0.02	0.14	0.06	0.24	0.11	0.31	0.18	0.39	0.03	0.17	0.08	0.27
Had complication information (ref.)	0.46	0.50	0.54	0.50	0.34	0.47	0.51	0.50	0.14	0.34	0.53	0.50	0.80	0.40	0.85	0.35	0.55	0.50	0.49	0.50
No Complication information	0.54	0.50	0.46	0.50	0.39	0.49	0.35	0.48	0.16	0.36	0.22	0.41	0.20	0.40	0.15	0.35	0.45	0.50	0.51	0.50
*Don't know*	-	-	-	-	0.03	0.16	0.02	0.12	0.01	0.11	0.01	0.11	-	-	-	-	-	-	-	-
*Missing*	-	-	-	-	0.25	0.43	0.12	0.32	0.70	0.46	0.24	0.42	-	-	-	-	-	-	-	-
Health facility permit	0.17	0.37	0.21	0.41	0.16	0.37	0.22	0.41	0.20	0.40	0.10	0.30	0.07	0.26	0.05	0.22	-	-	-	-
Health facility money	0.84	0.37	0.72	0.45	0.70	0.46	0.59	0.49	0.53	0.50	0.41	0.49	0.60	0.49	0.39	0.49	-	-	-	-
Health facility distance	0.56	0.50	0.43	0.50	0.53	0.50	0.42	0.49	0.53	0.50	0.25	0.44	0.45	0.50	0.22	0.41	-	-	-	-
Health facility alone	0.17	0.38	0.17	0.38	0.30	0.46	0.27	0.45	0.28	0.45	0.10	0.31	0.23	0.42	0.11	0.32	-	-	-	-
Health facility attitude	0.00	0.00	0.00	0.00	-	-	-	-	-	-	-	-	0.32	0.47	0.34	0.47	-	-	-	-
Has insurance coverage (ref.)					-	-	-	-	-	-	-	-	0.68	0.47	0.66	0.47	-	-	-	-
Uninsured					-	-	-	-	-	-	-	-	0.32	0.47	0.34	0.47		-	-	-

### The wealth-based trend in probability of the use of reproductive health care services

**Fig. 1:**
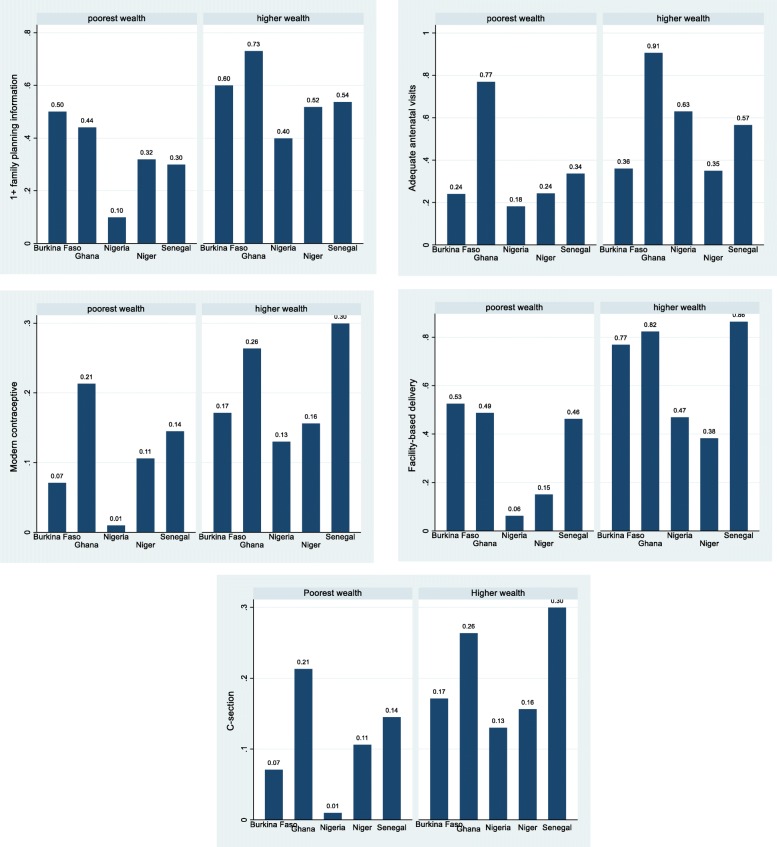
Mean of reproductive health care service use in the countries, by wealth category (weighted)

There were substantial differences between the low-wealth and high-wealth women in exposure to mass media information on family planning, the use of modern contraceptives, adequate antenatal care visits, and the use of facility-based childbirth services, and C-section at childbirth (see Figure 1). On average, 50% of women in the low-wealth group in Burkina Faso were exposed to mass media family planning information, in other countries this was less, e.g. only 10% in Nigeria. For the high-wealth category, at least 52% of women were exposed to family planning information, except in Nigeria (40%). The proportion of modern contraceptives use among women in the low-wealth group was generally low, namely 1% in Nigeria, 7% in Burkina Faso and 21% in Ghana, relative to 13%, 17% 26% among high-wealth group women in the same countries respectively. The wealth-based gap in the proportion of use of modern contraceptives was highest in Senegal - about 16 percentage points. Except for Ghana, the proportion with adequate antenatal care visits in the low-wealth group was relatively small; 18% in Nigeria, 24% in Niger and Burkina Faso, 34% in Senegal. The observed proportion of adequate visits among women in the high-wealth group ranged from 35% in Niger to 91% in Ghana. Among the low-wealth group, only 5% of women in Nigeria had facility-based childbirth, 15% in Niger and over 45% in the other countries. In the high-wealth category, at least 77% of women in Burkina Faso, Ghana, and Senegal had facility-based childbirth while Nigeria and Niger had lower proportions. About 1% of women in the low-wealth group living in Nigeria had a C-section compared with 21% of women in Ghana. In other countries, this ranged from 7% to 14%. On the other hand, of the women in the high-wealth group, 13% in Nigeria had a C-section birth, 30% in Senegal and 26% in Ghana.

As indicated above, there are substantial differences in the use of maternal health services between the five countries included in our study.

### Decomposition results

Tables 2, 3, 4, 5, and 6 presented the results of the decomposition analyses of exposure to family planning information via mass media, modern contraceptive use, adequate antenatal care visits, facility-based childbirth and C-sections (the outcome variables) among women in the low-wealth group and high-wealth group. In the upper part of these tables, the results of the probability of use, the difference or wealth gap, and the portion of that gap explained by observable characteristics are shown. The “explained” part is the proportion of the difference explained by the characteristics of women included in the analysis. If the low-wealth and high-wealth women had the same characteristics, then the “explained” portion would reduce the wealth-based gap in the outcome variable of interest. The lower part of these tables showed estimates of the contribution of socio-demographic characteristics to the explained portion of the gap. Negative estimates indicate that wealth differences in the independent variable increase the wealth gap (i.e. reduce the probability) in the use of reproductive health care. A positive estimate of the decomposition indicates the opposite. The key findings of the results are highlighted.

**Table 2:** Decomposition analysis of exposure to family planning information media source gaps

	Burkina Faso		Niger		Nigeria		Ghana		Senegal	
Pr(low-wealth)	0.498		0.318		0.096		0.450		0.308	
Pr(high-wealth)	0.604		0.517		0.399		0.734		0.539	
Difference	-0.107		-0.199		-0.302		-0.285		-0.231	
Total explained	-0.043		-0.103		-0.242		-0.190		-0.135	
% explained		40.2%		51.8%		80.2%		66.7%		58.4%
*Variable contribution*	Decomp.	%	Decomp.	%	Decomp.	%	Decomp.	%	Decomp.	%
Age	0.004**	-9.2	0.001**	-1.1	-0.001	0.4	0.002	-1.1	-0.006***	4.4
Children ever born	-0.008*	18.5	-0.000	0.0	-0.004	1.7	-0.013	6.9	-0.013**	9.6
No education	-0.029***	66.9	-0.019***	18.3	-0.125***	51.6	-0.070***	36.9	-0.044***	32.6
1^0^ education	0.013***	-30.0	0.004*	-3.5	0.011***	-4.5	-0.001*	0.5	0.008**	-5.9
Rural	-0.029***	66.9	-0.033***	32.2	-0.046***	19.0	-0.003	1.6	-0.023***	17.0
No religion	0.000	0.0	-	-			-	-	-	-
Islam	-0.002	4.6	-	-	0.016***	-6.6	0.001	-0.5	0.000	0.0
Traditional	0.001	-2.3	-	-	0.000	0.0	-0.004	2.1	-	-
Animist	-	0.0	-	-			-	-	-0.004***	3.0
No partner	0.000	0.0	0.000	-0.1	0.001*	-0.4	0.003	-1.6	0.004***	-3.0
No Complication information	0.000	2.3	-0.033***	32.3	-0.054***	22.3	-0.002	1.1	0.004***	-3.0
Don’t know			-0.000	0.1	0.000	0.0				
Missing			-0.004	3.4	-0.018***	7.4				
Health facility permit	-0.001	16.1	-0.003**	2.6	-0.001	0.4	0.000	0.0	-	-
Health facility money	-0.007***	13.8	-0.002	1.9	-0.003***	1.2	-0.013***	6.9	-	-
Health facility distance	-0.006***	0.0	-0.005***	5.0	-0.002	0.8	-0.005	2.6	-	-
Health facility alone	0.000	0.0	0.001***	-1.4	0.002	-0.8	0.001	-0.5	-	-
Health facility attitude	-	-	-	-	0.006***	-2.5	-	-	-	-
Uninsured	-	-	-	-			0.001*	-0.5	-	-
Cascades	-0.007***	16.1	-	-			-	-	-	-
Centre	-0.015***	34.6	-	-			-	-	-	-
Centre Est	-0.003***	6.9	-	-			-	-	-	-
Centre Nord	0.001**	-2.3	-	-			-	-	-	-
Centre Ouest	0.000	0.0	-	-			-	-	-	-
Centre Sud	-0.002***	4.6	-	-			-	-	-	-
Est	0.033***	-76.1	-	-			-	-	-	-
Hauts Basins	-0.013***	30.0	-	-			-	-	-	-
Nord	-0.003***	6.9	-	-			-	-	-	-
Plateau Central	-0.009***	20.8	-	-			-	-	-	-
Sahel	0.025***	-57.7	-	-			-	-	-	-
Sud Ouest	0.014***	-32.3	-	-			-	-	-	-
Agadez	-	-	-0.000	0.4			-	-	-	-
Diffa	-	-	0.002***	-2.0			-	-	-	-
Dosso	-	-	0.003*	-2.9			-	-	-	-
Maradi	-	-	-0.002	1.7			-	-	-	-
Tahoua	-	-	-0.005	4.7			-	-	-	-
Tillaberi	-	-	-0.007***	7.0			-	-	-	-
Zinder	-	-	-0.002	1.7			-	-	-	-
North Central	-	-	-	-	0.016***	-6.6	-	-	-	-
North East	-	-	-	-	-0.027***	11.1	-	-	-	-
North West	-	-	-	-	-0.043***	17.7	-	-	-	-
South East	-	-	-	-	0.014***	-5.8	-	-	-	-
South South	-	-	-	-	0.016***	-6.6	-	-	-	-
Western	-	-	-	-	-	-	-0.015***	7.9	-	-
Central	-	-	-	-	-	-	-0.017***	9.0	-	-
Volta	-	-	-	-	-	-	-0.001	0.5	-	-
Eastern	-	-	-	-	-	-	0.003*	-1.6	-	-
Greater Accra	-	-	-	-	-	-	-0.019***	10.0	-	-
Brong Ahafo	-	-	-	-	-	-	-0.004***	2.1	-	-
Northern	-	-	-	-	-	-	-0.001	0.5	-	-
Upper East	-	-	-	-	-	-	-0.034**	17.9	-	-
Upper West	-	-	-	-	-	-	0.001	-0.5	-	-
Ziguinchor	-	-	-	-	-	-	-	-	0.005***	-3.7
Diourbel	-	-	-	-	-	-	-	-	0.010***	-7.4
SaintLouis	-	-	-	-	-	-	-	-	0.001*	-0.7
Tambacounda	-	-	-	-	-	-	-	-	-0.021***	15.5
Kaolack	-	-	-	-	-	-	-	-	-0.004***	3.0
This	-	-	-	-	-	-	-	-	0.000	0.0
Louga	-	-	-	-	-	-	-	-	-0.008***	5.9
Fatick	-	-	-	-	-	-	-	-	-0.004***	3.0
Kolda	-	-	-	-	-	-	-	-	-0.020***	14.8
Matam	-	-	-	-	-	-	-	-	0.000	0.0
Kaffrine	-	-	-	-	-	-	-	-	-0.014**	10.4
Kedougou	-	-	-	-	-	-	-	-	-0.002***	1.5
Sedhiou	-	-	-	-	-	-	-	-	-0.004***	3.0
N	9831		7645		19911		4147		8839	

* *p*<0.05 ** *p*<0.01 *** *p*<0.001

**Table 3:** Decomposition of modern contraceptives use gaps

	Burkina Faso		Niger		Nigeria		Ghana		Senegal	
Pr(low-wealth)	0.077		0.106		0.009		0.218		0.148	
Pr(high-wealth)	0.176		0.156		0.130		0.265		0.302	
Difference	-0.099		-0.050		-0.121		-0.047		-0.154	
Total explained	-0.060		-0.053		-0.107		-0.014		-0.090	
% explained		60.3%		106.0%^a^		88.1%		30.6%		58.2%
*Variable contribution*	Decomp.	%	Decomp.	%	Decomp.	%	Decomp.	%	Decomp.	%
Age	0.000	0.0	-0.000	0.80	0	0.00	-0.003	20.84	0.001	-1.11
Children ever born	0.007*	-11.7	0.004	-7.50	0.011***	-10.30	0.026***	-180.64	0.025***	-27.84
No education	-0.031***	51.9	-0.015***	28.90	-0.050***	46.90	-0.023*	159.80	-0.037***	41.20
1^0^ education	0.006***	-10.1	0.003**	-6.50	0.001	-0.90	-0.000	0.00	0.003	-3.34
Rural	-0.024***	40.2	-0.028***	52.80	-0.012***	11.30	0.010	-69.48	-0.040***	44.54
No religion	0.000	0.0	-	-	-	-	-	-	-	-
Islam	0.004***	-6.7	-	-	-0.018***	16.90	-0.004	27.79	0.000	0.00
Traditional	-0.008***	13.4	-	-	-0.000*	0.00	0.003	-20.84		
Animist			-	-	-	-	-	-	-0.000	0.00
No partner	0.002***	-3.4	0.001***	-2.00	0.000	0.00	0.001	-6.95	0.004***	-4.45
No Complication information	-0.002**	3.4	-0.000	0.70	-0.003	2.80	0.001	-6.95	0.002*	-2.23
Don’t know	-	-	0.000	-0.90	0.000	0.00	-	-	-	-
Missing	-	-	-0.007***	13.00	-0.014***	13.10	-	-	-	-
Health facility permit	-0.000	0.0	0.000	-0.30	-0.002**	1.90	0.000	0.00	-	-
Health facility money	-0.003*	5.0	0.001	-2.10	0.000	0.00	-0.003	20.84	-	-
Health facility distance	-0.002*	3.4	-0.002	4.40	-0.002	1.90	-0.009*	62.53	-	-
Health facility alone	-0.000	0.0	-0.000	0.90	-0.002	1.90	0.002	-13.90	-	-
Health facility attitude	-	-	-	-	0.001	-0.90	-	-	-	-
Uninsured	-	-	-	-	-	-	-0.001*	6.95	-	-
Cascades	-0.002**	3.4	-	-	-	-	-	-	-	-
Centre	-0.008**	13.4	-	-	-	-	-	-	-	-
Centre Est	0.001	-1.7	-	-	-	-	-	-	-	-
Centre Nord	-0.000	0.0	-	-	-	-	-	-	-	-
Centre Ouest	-0.000	0.0	-	-	-	-	-	-	-	-
Centre Sud	0.000	0.0	-	-	-	-	-	-	-	-
Est	0.008*	-13.4	-	-	-	-	-	-	-	-
Hauts Basins	-0.010***	16.8	-	-	-	-	-	-	-	-
Nord	-0.000	0.0	-	-	-	-	-	-	-	-
Plateau Central	-0.000	0.0	-	-	-	-	-	-	-	-
Sahel	0.001	-1.7	-	-	-	-	-	-	-	-
Sud Ouest	0.002	-3.4	-	-	-	-	-	-	-	-
Agadez	-	-	0.000	-0.10	-	-	-	-	-	-
Diffa	-	-	0.000	-0.50	-	-	-	-	-	-
Dosso	-	-	-0.000	0.60	-	-	-	-	-	-
Maradi	-	-	-0.001	2.20	-	-	-	-	-	-
Tahoua	-	-	-0.006**	11.70	-	-	-	-	-	-
Tillaberi	-	-	-0.000	0.50	-	-	-	-	-	-
Zinder	-	-	-0.002	3.60	-	-	-	-	-	-
North Central	-	-	-	-	0.000	0.00	-	-	-	-
North East	-	-	-	-	-0.009***	8.40	-	-	-	-
North West	-	-	-	-	-0.016***	15.00	-	-	-	-
South East	-	-	-	-	0.004***	-3.80	-	-	-	-
South South	-	-	-	-	0.004***	-3.80	-	-	-	-
Western	-	-	-	-	-	-	-0.005	34.74	-	-
Central	-	-	-	-	-	-	-0.012***	83.37	-	-
Volta	-	-	-	-	-	-	0.001*	-6.95	-	-
Eastern	-	-	-	-	-	-	-0.002	13.90	-	-
Greater Accra	-	-	-	-	-	-	-0.004	27.79	-	-
Brong Ahafo	-	-	-	-	-	-	0.003**	-20.84	-	-
Northern	-	-	-	-	-	-	-0.029*	201.48	-	-
Upper East	-	-	-	-	-	-	0.027*	-187.59	-	-
Upper West	-	-	-	-	-	-	0.007	-48.63	-	-
Ziguinchor	-	-	-	-	-	-	-	-	0.002***	-2.23
Diourbel	-	-	-	-	-	-	-	-	0.010***	-11.14
SaintLouis	-	-	-	-	-	-	-	-	0.000	0.00
Tambacounda	-	-	-	-	-	-	-	-	-0.014***	15.59
Kaolack	-	-	-	-	-	-	-	-	-0.005***	5.57
This	-	-	-	-	-	-	-	-	0.000	0.00
Louga	-	-	-	-	-	-	-	-	-0.001	1.11
Fatick	-	-	-	-	-	-	-	-	-0.002*	2.23
Kolda	-	-	-	-	-	-	-	-	-0.019***	21.16
Matam	-	-	-	-	-	-	-	-	0.000	0.00
Kaffrine	-	-	-	-	-	-	-	-	-0.013***	14.48
Kedougou	-	-	-	-	-	-	-	-	-0.001**	1.11
Sedhiou	-	-	-	-	-	-	-	-	-0.004***	4.45
N	9838		7654		19959		4147		8839	

* *p*<0.05 ** *p*<0.01 *** *p*<0.001

^a^ The model explains 100% of the gap; the rest is noise due to the fact that the unexplained portion is negative.

**Table 4:** Decomposition of adequate antenatal visits gaps

	Burkina Faso		Niger		Nigeria		Ghana		Senegal	
Pr(low-wealth)	0.268		0.244		0.183		0.816		0.369	
Pr(high-wealth)	0.374		0.350		0.630		0.923		0.575	
Difference	-0.106		-0.106		-0.447		-0.106		-0.206	
Total explained	-0.068		-0.026		-0.182		-0.096		-0.129	
% explained		64.2%		24.6%		40.8%		90.6%		62.6%
*Variable contribution*	Decomp.	%	Decomp.	%	Decomp.	%	Decomp.	%	Decomp.	%
Age	0.003	-4.4	0.001	-3.2	-0.003**	1.6	0.010	-10.5	-0.011***	8.5
Children ever born	-0.013***	19.1	-0.002*	9.2	-0.011***	6.0	-0.026**	27.2	-0.041***	31.8
No education	-0.022***	32.4	-0.018***	68.4	-0.047***	25.8	-0.022*	23.0	-0.031***	24.0
1^0^ education	0.008***	-11.8	0.007***	-25.2	0.012***	-6.6	0.001	-1.1	0.009**	-7.0
Rural	-0.003	4.4	-0.006	24.3	-0.010*	5.5	-0.014*	14.7	-0.029***	22.5
No religion	0.000	0.0	-	-			-	-	-	-
Islam	0.004**	-5.9	-	-	0.001	-0.5	0.003	-3.1	0.000	0.0
Traditional	-0.009***	13.3	-	-	0.000	0.0	-0.004	4.2	-	-
Animist	-	-	-	-	-	-	-	-	0.000	0.0
No partner	0.000	0.0	-0.000	0.1	0.000	0.0	0.001	-1.1	0.005***	-3.9
No Complication information	0.000	0.0	-0.005	17.4	-0.028***	15.4	-0.001	1.1	0.005***	-3.9
Don’t know	-	-	-0.000	0.8	0.000	0.0	-	-	-	-
Missing	-	-	0.000	0.0	0.000	0.0	-	-	-	-
Health facility permit	-0.003**	**4.4**	-0.001	4.7	-0.001	0.5	0.001	-1.1	-	-
Health facility money	-0.002	2.9	-0.002	6.6	-0.005**	2.7	-0.005	5.2	-	-
Health facility distance	-0.004***	**5.9**	-0.004*	14.5	-0.023***	12.6	0.005	-5.2	-	-
Health facility alone	0.000	0.0	0.000	-0.4	0.005	-2.7	-0.011**	11.5	-	-
Health facility attitude	-	-	-	-	0.001	-0.5	-	-	-	-
Uninsured	-	-	-	-	-	-	0.002	-2.1	-	-
Cascades	0.000	0.0	-	-	-	-	-	-	-	-
Centre	-0.013***	19.1	-	-	-	-	-	-	-	-
Centre Est	-0.006***	8.8	-	-	-	-	-	-	-	-
Centre Nord	0.000	0.0	-	-	-	-	-	-	-	-
Centre Ouest	0.000	0.0	-	-	-	-	-	-	-	-
Centre Sud	-0.001**	1.5	-	-	-	-	-	-	-	-
Est	0.001	-1.5	-	-	-	-	-	-	-	-
Hauts Basins	0.002	-2.9	-	-	-	-	-	-	-	-
Nord	0.000	0.0	-	-	-	-	-	-	-	-
Plateau Central	-0.002*	2.9	-	-	-	-	-	-	-	-
Sahel	-0.016***	23.6	-	-	-	-	-	-	-	-
Sud Ouest	0.007**	-10.3	-	-	-	-	-	-	-	-
Agadez	-	-	-0.000	0.1	-	-	-	-	-	-
Diffa	-	-	0.000	-1.7	-	-	-	-	-	-
Dosso	-	-	0.003*	-12.3	-	-	-	-	-	-
Maradi	-	-	-0.001	3.1	-	-	-	-	-	-
Tahoua	-	-	-0.004	15.9	-	-	-	-	-	-
Tillaberi	-	-	0.002**	-7.7	-	-	-	-	-	-
Zinder	-	-	0.004	-14.5	-	-	-	-	-	-
North Central	-	-	-	-	0.025***	-13.7	-	-	-	-
North East	-	-	-	-	-0.042***	23.0	-	-	-	-
North West	-	-	-	-	-0.079***	43.3	-	-	-	-
South East	-	-	-	-	0.005*	-2.7	-	-	-	-
South South	-	-	-	-	0.017***	-9.3	-	-	-	-
Western	-	-	-	-	-	-	0.005	-5.2	-	-
Central	-	-	-	-	-	-	0.006	-6.3	-	-
Volta	-	-	-	-	-	-	0.000	0.0	-	-
Eastern	-	-	-	-	-	-	0.010***	-10.5	-	-
Greater Accra	-	-	-	-	-	-	0.019**	-19.9	-	-
Brong Ahafo	-	-	-	-	-	-	0.000	0.0	-	-
Northern	-	-	-	-	-	-	-0.075***	78.5	-	-
Upper East	-	-	-	-	-	-	0.002	-2.1	-	-
Upper West	-	-	-	-	-	-	-0.002	2.1	-	-
Ziguinchor	-	-	-	-	-	-	-	-	0.000	0.0
Diourbel	-	-	-	-	-	-	-	-	0.006**	-4.7
SaintLouis	-	-	-	-	-	-	-	-	0.001*	-0.8
Tambacounda	-	-	-	-	-	-	-	-	0.000	0.0
Kaolack	-	-	-	-	-	-	-	-	-0.001	0.8
This	-	-	-	-	-	-	-	-	0.004	-3.1
Louga	-	-	-	-	-	-	-	-	-0.006***	4.7
Fatick	-	-	-	-	-	-	-	-	-0.002*	1.6
Kolda	-	-	-	-	-	-	-	-	-0.023***	17.8
Matam	-	-	-	-	-	-	-	-	0.001**	-0.8
Kaffrine	-	-	-	-	-	-	-	-	-0.011*	8.5
Kedougou	-	-	-	-	-	-	-	-	-0.001	0.8
Sedhiou	-	-	-	-	-	-	-	-	-0.003*	2.3
N	9831		7619		19427		4127		8690	

* *p*<0.05 ** *p*<0.01 *** *p*<0.001

**Table 5:** Decomposition of use of facility-based childbirth services gaps

	Burkina Faso		Niger		Nigeria		Ghana		Senegal	
Pr(low-wealth)	0.581		0.151		0.063		0.514		0.499	
Pr(high-wealth)	0.793		0.382		0.469		0.833		0.874	
Difference	-0.212		-0.232		-0.407		-0.320		-0.375	
Total explained	-0.142		-0.156		-0.369		-0.244		-0.179	
% explained		67.0%		67.2%		90.8%		76.3%		47.7%
*Variable* *contribution*	Decomp.	%	Decomp.	%	Decomp.	%	Decomp.	%	Decomp.	%
Age	0.000	0.0	0.001	-0.9	-0.006***	1.6	0.007**	-2.9	-0.001	0.6
Children ever born	-0.008*	5.6	-0.006***	3.9	-0.015***	4.1	-0.047***	19.2	-0.031***	17.3
No education	-0.018***	12.7	-0.022***	14.4	-0.083***	22.5	-0.053***	21.7	-0.029***	16.2
1^0^ education	0.006	-4.2	0.003	-1.9	0.008***	-2.2	0.002	-0.8	0.008*	-4.5
Rural	-0.020***	14.1	-0.069***	44.3	-0.036***	9.7	-0.068***	27.8	-0.043***	24.0
No religion	0.000	0.0	-	-	-	-	-	-	-	-
Islam	0.007***	-4.9	-	-	-0.026***	7.0	0.003	-1.2	0.000	0.0
Traditional	-0.016***	11.3	-	-	0.000	0.0	-0.004	1.6	-	-
Animist	-	-	-	-			-	-	-0.001	0.6
No partner	0.000	0.0	-0.000	0.1	0.001**	-0.3	0.000	0.0	0.001	-0.6
No Complication information	-0.008***	5.6	-0.013***	8.6	-0.024***	6.5	-0.003**	1.2	0.003***	-1.7
Don't know	-	-	-0.000	0.1	0.000	0.0	-	-	-	-
Missing	-	-	-0.025***	15.7	-0.104***	28.2	-	-	-	-
Health facility permit	-0.004***	2.8	-0.002*	1.1	-0.001	0.3	-0.001	0.4	-	-
Health facility money	0.000	0.0	0.001	-0.3	-0.003***	0.8	0.006	-2.5	-	-
Health facility distance	-0.021***	14.8	-0.013***	8.2	-0.008***	2.2	-0.008	3.3	-	-
Health facility alone	0.000	0.0	-0.000	0.2	-0.002	0.5	-0.006	2.5	-	-
Health facility attitude	-	-	-	-	0.003**	-0.8	-	-	-	-
Uninsured	-	-	-	-	-	-	0.002*	-0.8	-	-
Cascades	-0.002*	1.4	-	-	-	-	-	-	-	-
Centre	-0.009***	6.3	-	-	-	-	-	-	-	-
Centre Est	-0.005***	3.5	-	-	-	-	-	-	-	-
Centre Nord	0.000	0.0	-	-	-	-	-	-	-	-
Centre Ouest	0.000	0.0	-	-	-	-	-	-	-	-
Centre Sud	-0.003***	2.1	-	-	-	-	-	-	-	-
Est	-0.008**	5.6	-	-	-	-	-	-	-	-
Hauts Basins	0.000	0.0	-	-	-	-	-	-	-	-
Nord	0.001	-0.7	-	-	-	-	-	-	-	-
Plateau Central	-0.004***	2.8	-	-	-	-	-	-	-	-
Sahel	-0.024***	16.9	-	-	-	-	-	-	-	-
Sud Ouest	-0.005*	3.5	-	-	-	-	-	-	-	-
Agadez	-	-	0.000	-0.1	-	-	-	-	-	-
Diffa	-	-	-0.001	0.4	-	-	-	-	-	-
Dosso	-	-	0.001	-0.8	-	-	-	-	-	-
Maradi	-	-	-0.001	0.7	-	-	-	-	-	-
Tahoua	-	-	-0.005	3.1	-	-	-	-	-	-
Tillaberi	-	-	0.000	-0.2	-	-	-	-	-	-
Zinder	-	-	-0.005*	3.5	-	-	-	-	-	-
North Central	-	-	-	-	0.002	-0.5	-	-	-	-
North East	-	-	-	-	-0.029***	7.8	-	-	-	-
North West	-	-	-	-	-0.056***	15.2	-	-	-	-
South East	-	-	-	-	-0.005***	1.4	-	-	-	-
South South	-	-	-	-	0.013***	-3.5	-	-	-	-
Western	-	-	-	-	-	-	0.011***	-4.5	-	-
Central	-	-	-	-	-	-	0.018***	-7.4	-	-
Volta	-	-	-	-	-	-	0.000	0.0	-	-
Eastern	-	-	-	-	-	-	0.005***	-2.0	-	-
Greater Accra	-	-	-	-	-	-	0.003	-1.2	-	-
Brong Ahafo	-	-	-	-	-	-	0.000	0.0	-	-
Northern	-	-	-	-	-	-	-0.117***	47.9	-	-
Upper East	-	-	-	-	-	-	0.006	-2.5	-	-
Upper West	-	-	-	-	-	-	0.000	0.0	-	-
Ziguinchor	-	-	-	-	-	-	-	-	0.001	-0.6
Diourbel	-	-	-	-	-	-	-	-	0.000	0.0
SaintLouis	-	-	-	-	-	-	-	-	0.000	0.0
Tambacounda	-	-	-	-	-	-	-	-	-0.023***	12.8
Kaolack	-	-	-	-	-	-	-	-	0.000	0.0
This	-	-	-	-	-	-	-	-	-0.007**	3.9
Louga	-	-	-	-	-	-	-	-	0.000	0.0
Fatick	-	-	-	-	-	-	-	-	0.000	0.0
Kolda	-	-	-	-	-	-	-	-	-0.038***	21.2
Matam	-	-	-	-	-	-	-	-	0.002*	-1.1
Kaffrine	-	-	-	-	-	-	-	-	-0.012**	6.7
Kedougou	-	-	-	-	-	-	-	-	-0.002***	1.1
Sedhiou	-	-	-	-	-	-	-	-	-0.005***	2.8
N	9832		7652		19919		4146		8839	

* *p*<0.05 ** *p*<0.01 *** *p*<0.001

**Table 6:** Decomposition of having C-section at childbirth gaps

	Burkina Faso		Niger		Nigeria		Ghana		Senegal	
Pr(low-wealth)	0.010		0.004		0.005		0.050		0.026	
Pr(high-wealth)	0.027		0.019		0.029		0.162		0.073	
Difference	-0.017		-0.016		-0.024		-0.112		-0.048	
Total explained	-0.016		-0.010		-0.025		-0.106		-0.038	
% explained		94.12%		62.50%		102.77% ^a^		94.64%		79.17%
*Variable contribution*	Decomp.	%	Decomp.	%	Decomp.	%	Decomp.	%	Decomp.	%
Age	0.002	-12.5	0.000	-3.2	-0.001	4.1	0.001	-0.9	-0.005	13.2
Children ever born	-0.003	18.8	-0.000	2.8	-0.003**	12.2	-0.034***	32.1	-0.011***	28.9
No education	-0.002	12.5	-0.003*	26.3	-0.006**	24.4	-0.022**	20.8	-0.006*	15.8
1^0^ education	0.000	0.0	0.001	-5.6	0.000	0.0	-0.001	0.9	0.002*	-5.3
Rural	-0.008***	50.0	-0.005*	48.8	-0.004***	16.2	-0.017**	16.0	-0.008*	21.1
No religion	0.000	0.0	-	-	-	-	-	-	-	-
Islam	0.000	0.0	-	-	-0.003**	12.2	0.005	-4.7	0.000	0.0
Traditional	0.000	0.0	-	-	0.000	0.0	0.006	-5.7	-	-
Animist	0.000		-	-	-	-	0.000	0.0	0.000	0.0
No partner	-	0.0	-0.000	0.1	0.000	0.000	-	-	-0.001*	2.6
No Complication information Don't know	0.000 -	0.0 -	-0.000 0.001	2.7 -14.3	-0.004*** 0.000	16.2 0.0	-0.003* -	2.8 -	0.002** -	-5.3 -
Missing	-	-	-0.001	6.2	-0.002	8.1	-	-	-	-
Health facility permit	0.000	0.0	-0.000	2.2	0.000	0.0	-0.001	0.9	-	-
Health facility money	0.000	0.0	-0.001	6.4	-0.001*	4.1	-0.002	1.9	-	-
Health facility distance	0.000	0.0	-0.000	0.5	0.000	0.0	0.002	-1.9	-	-
Health facility alone	0.000	0.0	-0.000	1.5	-0.001	4.1	0.000	0.0	-	-
Health facility attitude	-	-	-	-	0.000	0.0	-	-	-	-
Uninsured	-	-	-	-	-	-	0.000	0.0	-	-
Cascades	0.000	0.0	-	-	-	-	-	-	-	-
Centre	-0.003	18.8	-	-	-	-	-	-	-	-
Centre Est	0.000	0.0	-	-	-	-	-	-	-	-
Centre Nord	0.000	0.0	-	-	-	-	-	-	-	-
Centre Ouest	0.000	0.0	-	-	-	-	-	-	-	-
Centre Sud	0.000	0.0	-	-	-	-	-	-	-	-
Est	-0.001	6.3	-	-	-	-	-	-	-	-
Hauts Basins	0.000	0.0	-	-	-	-	-	-	-	-
Nord	0.000	0.0	-	-	-	-	-	-	-	-
Plateau Central	0.000	0.0	-	-	-	-	-	-	-	-
Sahel	-0.001**	6.3	-	-	-	-	-	-	-	-
Sud Ouest	0.000	0.0	-	-	-	-	-	-	-	-
Agadez	-	-	0.000	-0.9	-	-	-	-	-	-
Diffa	-	-	0.000	-2.1			-	-	-	-
Dosso	-	-	0.000	-3.4	-	-	-	-	-	-
Maradi	-	-	-0.000	0.8	-	-	-	-	-	-
Tahoua	-	-	-0.003	25.6			-	-	-	-
Tillaberi	-	-	-0.000	3.4	-	-	-	-	-	-
Zinder	-	-	-0.000	2.1	-	-	-	-	-	-
North Central	-	-	-	-	0.000	0.0	-	-	-	-
North East	-	-	-	-	0.002	-8.1	-	-	-	-
North West	-	-	-	-	-0.001	4.1	-	-	-	-
South East	-	-	-	-	0.000	0.0	-	-	-	-
South South	-	-	-	-	-0.001	4.1	-	-	-	-
Western	-	-	-	-	-	-	0.000	0.0	-	-
Central	-	-	-	-	-	-	0.000	0.0	-	-
Volta	-	-	-	-	-	-	-0.002**	1.9	-	-
Eastern	-	-	-	-	-	-	0.000	0.0	-	-
Greater Accra	-	-	-	-	-	-	0.004	-3.8	-	-
Brong Ahafo	-	-	-	-	-	-	-0.002*	1.9	-	-
Northern	-	-	-	-	-	-	-0.027***	25.5	-	-
Upper East	-	-	-	-	-	-	-0.012	11.3	-	-
Upper West	-	-	-	-	-	-	-0.003	2.8	-	-
Ziguinchor	-	-	-	-	-	-	-	-	0.001	-2.6
Diourbel	-	-	-	-	-	-	-	-	-0.001	2.6
SaintLouis	-	-	-	-	-	-	-	-	0.000	0.0
Tambacounda	-	-	-	-	-	-	-	-	-0.002	5.3
Kaolack	-	-	-	-	-	-	-	-	-0.001*	2.6
This	-	-	-	-	-	-	-	-	0.002*	-5.3
Louga	-	-	-	-	-	-	-	-	0.000	0.0
Fatick	-	-	-	-	-	-	-	-	-0.001*	2.6
Kolda	-	-	-	-	-	-	-	-	-0.005*	13.2
Matam	-	-	-	-	-	-	-	-	0.000	0.0
Kaffrine	-	-	-	-	-	-	-	-	-0.003	7.9
Kedougou	-	-	-	-	-	-	-	-	0.000	0.0
Sedhiou	-	-	-	-	-	-	-	-	-0.001*	2.6
N	9838		7654		19959		4147		8750	

* *p*<0.05 ** *p*<0.01 *** *p*<0.001

^a^ The model explains 100% of the gap; the rest is noise due to the fact that the unexplained portion is negative.

#### Exposure to family planning information via mass media

Table 2 shows that the wealth-based gap in the probability of exposure to mass media messages was largest in Nigeria (0.302) and smallest in Burkina Faso (0.107). The included variables explain about 80.2% (Nigeria), 66% (Ghana), 55% (Senegal), 51.8% (Niger) and 40% (Burkina Faso) of this gap. The contribution of the number of children ever born by a woman to the gap was significant in Burkina Faso and Senegal and contributed about 18% and 9% respectively. Money problems in visiting a health facility significantly increased the gap in Burkina Faso, Ghana, and Nigeria respectively. Distance problems contributed to the gap in Burkina Faso and Niger. Getting permission to get medical help for self and attitude of health facility workers increased the wealth gap only in Nigeria. Lack of pregnancy complication information increased the gap by as much as 32% in Niger, 22% in Nigeria, but decreased the gap in Senegal 3%. The Centre region of Burkina Faso, the Tillaberi in Niger, North East in Nigeria, Upper East in Ghana, and Tambacounda region. Senegal has the largest contribution to the wealth gaps observed.

#### Modern contraceptive use

Table 3 indicates that the wealth-based gap in the probability of modern contraceptive use was largest in Senegal (0.154) followed by Nigeria (0.121) and lowest in Burkina Faso (0.099) while Niger and Ghana had wealth gaps of 0.050 and 0.049 respectively. The disparity in modern contraceptive use explained by observable characteristics was about 88% of the wealth gap in Nigeria, 60% in Burkina Faso, 58% in Senegal, 30% in Ghana. The observed characteristics explained the entire low/high-wealth gap in modern contraceptive use in Niger. There might be some unexplained factor that works in the opposite direction. Woman’s number of children ever born reduced the wealth gap in the use of modern contraceptives in all countries. Of the problems associated with seeking care at a health facility, the distance was most prominent and had the effect of increasing the wealth gap in the probability of use of modern contraceptives in Ghana by around 62% and less in Senegal. For health facility, money problems explained the wealth gap in Burkina Faso and permission to get medical help for self only in Nigeria. Regions that considerably increased the wealth gaps were Hauts Basins in Burkina Faso, Tahoua in Niger, North East region in Nigeria, Northern region in Ghana as well as Kolda region in Senegal. None of the regions in Niger reduced the wealth gap in modern contraceptives use.

#### Adequate antenatal care visits

Table 4 shows that low-wealth women in Burkina Faso (0.268), Niger (0.244), Nigeria (0.183) and Senegal (0.369) have relatively little chances of receiving an adequate number of antenatal visits. But relatively higher in Ghana (0.816). The wealth-based gap in the probability of having adequate antenatal visits was 0.206 in Senegal and 0.447 in Nigeria. Of this disparity in an adequate number of antenatal visits, characteristics included in the model explained as little as 25% in Niger and as much as 91% in Ghana. In Burkina Faso, Senegal, and Nigeria, the explained portion was 64%, 62%, and 40% respectively. The number of children ever born by a woman increased the wealth gap by not less than 5% in all five countries. Regarding health facility seeking problems, the problem of permission to get medical help self significantly increased the wealth-based gap in adequate antenatal care use in Burkina Faso only. Money problems that prevent a health facility visit explained the wealth gap in having an adequate number of antenatal care visits in Nigeria, distance problems explained the wealth gap in Burkina Faso and Nigeria, while the problem of going alone to a health facility did so only in Ghana. Regional contributions to increased disparities were explained by seven regions in Senegal (mostly the Kolda region), and five in Burkina Faso (mostly the Sahel region). Two regions in Nigeria and one region in both Niger and Ghana increased the wealth-gap in adequate antenatal care visits.

#### Facility-based childbirth

Table 5 reveals that the wealth-based gap in the probability of having facility-based childbirth was 0.407 in Nigeria, 0.375 in Senegal, 0.320 in Ghana. The wealth gap was of smaller magnitude in Niger and Burkina Faso. Across the five countries, the characteristics included for each country model explained between 47% in Senegal and 90% in Nigeria of the observed wealth gap in facility-based childbirth. The wealth gap in facility-based childbirth also significantly increased by the number of children ever born by a woman across the countries and explains as much as 19% in Ghana and 17% in Senegal. The problem of needing permission to get medical help for self was significant in Burkina Faso and Niger, the problem of money to visit a health facility was significant only in Nigeria, and distance to the health facility was a significant problem in Burkina Faso, Niger, and Nigeria – all increasing the wealth gap. Regions, which most prominently contributed to the gap, were the Sahel in Burkina Faso, Zinder in Niger, North West region in Nigeria, Northern region in Ghana, and Kolda region in Senegal.

#### C-section childbirth

Table 6 shows that within the high-wealth group, women in Ghana had the highest probability of a C-section, 0.162, followed by Senegal, 0.073. Large wealth gaps in the probability of a C-section between women groups were observed in Ghana and Senegal. Burkina Faso, Niger and Nigeria had a relatively lower probability of a C-section gap between wealth groups, however, the probability of a C-section was also relatively similar in these countries. Of the wealth-based difference in C-section, the characteristics included in the model explained 94.6% of the variation in Ghana, 79.1% in Senegal, 94.1% in Burkina Faso and 62.5% in Niger. The characteristics included in the model explained the total low/high-wealth gap in C-section in Nigeria. The number of children ever born was statistically significant and increased the wealth gap in C-sections in Nigeria, Ghana, and Senegal. Significant health facility seeking characteristics that increased the wealth gap included money problems in Nigeria, Niger, and Ghana. Lack of pregnancy complication information increased the wealth gap in C-sections in Nigeria and Ghana. An increase in the wealth gap was significant only in the Sahel region in Burkina Faso and only the South-South region in Nigeria. No region in Niger contributed to the increased wealth gap but three regions were observed in Ghana and four in Senegal.

## Discussion

Our findings confirm the presence of wealth-based inequalities to the detriment of women in low-wealth households in the use of reproductive health care services. These results are in line with other studies, which have established the disadvantage of poor women in the use of reproductive health care services in sub-Saharan Africa [3, 5, 7]. The findings show that differences in characteristics between the two wealth groups explain a considerable part of the wealth gap in all five reproductive health care services we studied. More importantly, differences in the distribution of observed maternal characteristics contribute to the observed wealth-based inequalities in reproductive health care use in all five countries. There is evidence to suggest that the probability of the use of reproductive health care services is not parallel to the inequality gap. We find that the probability of women in low-wealth households to use reproductive health care services is higher in Burkina Faso for exposure to family planning information via mass media and facility-based childbirth services, while it is higher in Ghana for modern contraceptives, an adequate number of antenatal visits and C-Sections. However, we observe that the wealth-based inequalities in reproductive health care use are higher in other countries; in Nigeria for exposure to family planning information via mass media, an adequate number of antenatal visits and facility-based childbirth, while Senegal has a higher inequality gap in modern contraceptive use and C-section. This observation could be due to coverage of the cost of services by third parties [42].

We observe that 71% of the disparity in exposure to family planning information via mass media is explained by socio-demographic differences between women in the different wealth groups. This suggests that poorer women have characteristics that prevent them from receiving family planning messages. Socio-demographic characteristics such as religion, residence, and marital status negatively influence the exposure to family planning information, which has also been observed in other studies [11]. It can, therefore, be said that family planning information via mass media has not been well targeted to the poor population group, and does not address their peculiarities. For example, some religious practices encourage polygamy and do not encourage family planning programs [11]. Moreover, policies to disseminate information via mass media by governments, seldom address differentials in the characteristics of women or remain unimplemented. One such policy is Nigeria’s national reproductive health policy and strategy to promote reproductive health education through mass media [43, 44]. Alternatively, the urban reproductive health initiative in Senegal takes into consideration the influence of religion and attempts to fill the gaps in exposure to family planning information across urban dwellers [45, 46]. Population-wide policies are too broad and often benefit the better-off. Interventions, which do not promote fairness in access for most vulnerable groups, usually widen the inequality gaps.

Unsurprisingly, differences in maternal characteristics increase inequality and explain the observed gap in modern contraceptive use between the poorest and wealthier women. Though the average modern contraceptive use is appreciably low (Fig. 1), country policies have singled-out what is included in reproductive health care packages. In Ghana, for example, the NHIS which covers maternal care does not cover family planning services [30]. We observe a significant contribution of rural disparity in other countries but not in Ghana. Perhaps the community-based health planning and services are better able to address the problem of service availability in remote areas and rural districts of Ghana. There however seems to be only a provider-focused delivery strategy to increase the prevalence of modern contraceptive prevalence and so ignoring individual factors, such as distance, which explains as much as 62% of the disparity in the use of modern contraceptives in Ghana. The distance to a health facility is a problem because it is related to the indirect costs of transportation which are incurred when seeking modern contraceptives while these costs are not covered under any national health promotion scheme and thus, they constitute a burden for poor women. The association of distance and contraceptive use echoes earlier studies that indicate contraceptive use declines among women are five kilometers away from a health facility in Burkina Faso [47] and two kilometers away from a community-based health planning centers in the Upper East region of Ghana [48]. Further, the trend across regions suggests that in addition to the imbalanced distribution of resources, the region is relevant to understand the disparities in family planning services. Further research is needed to understand the sociocultural factors that cause regional disparities. Health inequalities are a result of a variety of demand-side as well as supply-side factors [49]. Such evidence could be valuable input for developing a policy to encourage not only the use of family planning commodities but all aspects of reproductive health care.

Moreover, our three indicators of maternal health utilization suggest that not all countries, which have abolished user fees for maternal care completely, have performed similarly in reducing the inequality between wealth groups. Nonetheless, it appears that in countries with complete fee exemption policies, that is, Ghana, Niger, and Senegal, the between wealth groups gaps in having an adequate number of antenatal care visits, facility-based childbirth and C-section are smaller. Although, comparatively, it seems that Senegal has not achieved as much as the other two countries which can allude to the fact that unlike Ghana and Niger, Senegal’s maternal care policy does not cover antenatal care [32]. From our findings, it appears that the poorest women remain unable to surmount other barriers that prevent them from using subsidized facility-based childbirth care. We find that the number of children ever born by a woman, the cost of transportation and cost at the point of use are significant barriers that deter women from the appropriate use of antenatal care services. These are also consistent with findings from previous studies [8, 15]. This is an indication that the abolishment of the user fee for maternal care services may contribute to wealth-based inequality in the use of these services.

The explanatory variables included for Nigeria explain more than 100% of the gap, indicating that the covariates explained more than the observed difference in rates of use. Thus, the unexplained gap in C-section between the poorest and wealthier women contributes positively to the gap. This finding suggests that the covariates included for poor women explain all the observed disparity in having a C-section. It is also likely that Nigeria’s removal of user fees policy for C-section through the national health insurance scheme - maternal and child health project has fostered more equitable access to obstetric health care services especially among women of lower economic status [28]. The overall coverage of the project, however, is low and fragmented [50]. Elimination of user fees for maternal care services like C-sections goes only a short way in alleviating the out-of-pocket costs incurred when seeking care and increase the wealth-based inequalities to the detriment of poorer households. Evidence of this is observed in the wealth gap observed in C-section between women in low-wealth and high wealth households in Ghana. Other studies have found that poorer women remain at a disadvantage of Ghana’s fee exemption policy for childbirth care despite the policy [16, 51]. In other countries with policies that also cover C-section, gaps in reproductive health care use between the wealth groups persist. For example, a user fee policy in Burkina Faso exempts the poorest women from fees in the case of C-section childbirth. There is also the free C-section policy in Senegal. Comparatively, the inequality gaps in C-section is lower in Burkina Faso than Senegal. This suggests that aside service fees, other barriers encountered by the poorest to use the subsidized services remain insurmountable. This finding is corroborated by other studies [19, 52]. A study in Senegal reveals that the physical distribution of facilities with C-section capabilities does not favor the grassroots women [32]. A study in Mali discussed that while richer households can raise emergency funds needed to access care services, the poorest households have much more difficulty doing so [53].

Our study indicates that other factors aside the cost of services at the point of use contributed to the inequality in the use of subsidized maternal health care. Cost of transport, as well as money spent on services at the health facility, are not always fully reimbursed under health schemes. A subpopulation study in Nigeria describing the free maternal health care intervention effect observes that the use of antenatal health care reduces with distance despite free maternal care policy [27]. Other studies clarify that money for health facility hinders women from profiting from the national maternal health care subsidy policy [15]. Another maternal characteristic, namely knowledge of pregnancy care, added to the disadvantage of poor women. The lack of information on pregnancy complications among poor women increases the gap in the use of reproductive health care, including adequate antenatal care visits. Information dissemination about the free maternal health childbirth is ineffective and disadvantaged women in poor households are unable to take benefit [27]. The problem of needing permission to get medical help for self adds to the disadvantage of the poorest households, which they have when needing reproductive health care services, including modern contraceptives and antenatal. Eliminating user fees could have impacted on women’s empowerment and their ability to get to a health facility. Studies have confirmed the existence of settings where women require spousal or family permission or escort to make use of reproductive care [54]. The number of children ever born by a woman in poor households increased the wealth-based gap. In addition to economic challenges when seeking care, women who have gone through childbirth would rely on previous experiences such as the negative attitude of health workers and travel difficulties when deciding on the use of reproductive health care [55, 56]. Regional disparities observed within all the countries implicate cultural differences or structural inadequacies in the availability and distribution of reproductive health care. Findings from other studies confirm that unfair allocation of resources contribute to geographical disparity, concluding that supply-side policies addressed at wealth inequalities in utilization of care are ineffective if they do not account for social determinants of health [41, 57].

For policy purposes, it is necessary to explore a practical and sustainable way to address wealth-based inequality in reproductive health care use. Governments in resource-poor West African countries, need to design reproductive health care programs, which target additional services for the poor. The strategies towards universal health coverage should capture not just everybody being covered but should pay attention to the allocation of health services to groups with the highest needs – typically poor groups. This will maximize the intervention impact and cost-effectiveness. Based on our findings, a policy targeting socio-demographic determinants of health to capture the differences in marital status, financial expenditure for accessing health services, knowledge of pregnancy care, and geographical location, will be preferred.

### Methodological considerations

This study has some limitations that need to be acknowledged. Many characteristics included in the study, are likely to be related to the use of reproductive health care but there are also other factors likely to be related to use, for example, health condition during pregnancy, which we could not control for. In addition, there are important observable and non-observable differences between service users and non-users which differ from a country to another. These factors could not be included given data constraints. We acknowledge that neither the poor nor the rich can use services that do not exist while our study does not analyze coverage gaps. We analyzed five countries in West Africa with surveys conducted in different years. Although this does not take away from the results or the recommendations, comparability must be done with caution. This paper does not address questions on the causes of poverty but rather touches on inequalities in the troublesome issues of social determinants of health. Lastly, cross-sectional survey data can only reveal association not necessarily a causal relationship between health outcomes and covariates.

## Conclusion

Using national-level data from Burkina Faso, Niger, Nigeria, Ghana and Senegal, the results of this study elucidate that women in households with low wealth are at a disadvantage despite reproductive health care policies to eliminate inequalities. Inequalities exist due to differences in the characteristics between the low wealth and wealthier groups of women. Evidence of this is in the substantial proportions of the explained gap by maternal characteristics. All countries analyzed have or are in the process of nationwide interventions to improve maternal health care. In all five countries, women of reproductive ages in the poorest households have lower chances of getting reproductive health care services. Although the contribution of these characteristics differs among countries, they remain relevant barriers to the use of reproductive health care services. With the introduction of targeted policies to mitigate the impact of these contributors, vulnerable women’s use of reproductive health care services could be increased at least to the level of their better-off counterparts in wealthier households. Such as engaging companions and provision of transportation to reproductive health care centers to mitigate the impact of permission problems when in need of medical help. Furthermore, communication strategies and the provision of family planning services that specifically target women in poor households need to be developed. These should be mindful of fertility preference, religion, residence, and marital status to increase access to family planning services. It is also important to expand family planning efforts to include reimbursement or fee exemption policies similar to maternal health care schemes to improve childbirth services for both aspects of reproductive health care.

## Supplementary information

**Additional file 1: Table S1.** Logit and decomposition results. **Table S2.** Sensitivity analysis by using the coefficients of the upper three quintiles against the lower two quintiles. **Table S3.** Summary of regional covariates included in the analysis. **Supplementary B**: Fairlie decomposition detailed.

## Data Availability

The data that support the findings of this study are available from the DHS program (https://www.dhsprogram.com/data/available-datasets.cfm) but restrictions apply to the availability of these data, which were used under license for the current study, and so are not publicly available. Data are however available from the authors upon reasonable request and with permission of the DHS program.
