# Supplementary material for: Inequalities in reproductive health care use in five West-African countries: A decomposition analysis of the wealth-based gaps
Source: Int J Equity Health. 2020 Mar 27;19:44. doi: 10.1186/s12939-020-01167-7 (PMC7099835; doi:10.1186/s12939-020-01167-7)
Supplement: Supplementary file 1 — Additional file 1: Table S1. Logit and decomposition results. Table S2. Sensitivity analysis by using the coefficients of the upper three quintiles against the lower two quintiles. Table S3. Summary of regional covariates included in the analysis. Supplementary B: Fairlie decomposition detailed. [file 12939_2020_1167_MOESM1_ESM.pdf]

ADDITIONAL FILE 1

Supplementary Table 1: Logit and decomposition results

Logit Regressions for Probability of reproductive health care service use in Burkina Faso

Family planning exposure

Logistic regression number of obs.	=	8091.000
LR chi2(26)	=	992.180
Prob > chi2	=	0.000
Log likelihood	=	-4935.081
Pseudo R2	=	0.091

Family planning exposure	Coef.	Std. Err.	z	P>z	95% Conf. interval	
Constant	1.126	0.214	5.250	0.000	0.706	1.547
Age	0.017	0.006	2.700	0.007	0.005	0.030
Children ever born	-0.043	0.020	-2.200	0.028	-0.082	-0.005
2^0 / 3^0 education (ref.)						
No education	-1.071	0.131	-8.180	0.000	-1.327	-0.814
1^0 education	-0.795	0.140	-5.670	0.000	-1.070	-0.521
Urban(ref.)						
rural	-0.747	0.079	-9.480	0.000	-0.901	-0.592
Catholic / protestant (ref.)						
No religion	-0.081	0.292	-0.280	0.783	-0.653	0.492
Islam	0.094	0.060	1.560	0.119	-0.024	0.212
Traditional / Other	0.032	0.119	0.270	0.790	-0.201	0.264
No partner	0.062	0.149	0.420	0.676	-0.230	0.354
No complication information	-0.023	0.051	-0.460	0.647	-0.123	0.076
Health facility permit	0.082	0.075	1.100	0.273	-0.065	0.228
Health facility money	-0.278	0.061	-4.600	0.000	-0.397	-0.160
Health facility distance	-0.255	0.056	-4.550	0.000	-0.365	-0.145
Health facility alone	0.039	0.081	0.480	0.633	-0.120	0.197
BoucledeMo~n (ref)						
Cascades	0.942	0.134	7.050	0.000	0.680	1.204
Centre	0.783	0.116	6.740	0.000	0.555	1.011
CentreEst	0.499	0.104	4.810	0.000	0.295	0.702
CentreNord	0.933	0.107	8.760	0.000	0.725	1.142
CentreOuest	-0.354	0.111	-3.180	0.001	-0.572	-0.136
CentreSud	1.201	0.130	9.250	0.000	0.946	1.455
Est	1.436	0.114	12.560	0.000	1.212	1.660
HautsBasins	1.062	0.101	10.500	0.000	0.864	1.260
Nord	0.912	0.108	8.470	0.000	0.701	1.122
Plateau-Central	1.689	0.135	12.540	0.000	1.425	1.953
Sahel	0.848	0.121	6.990	0.000	0.610	1.086
SudOuest	1.222	0.167	7.330	0.000	0.895	1.548

Modern contraceptive use

Logistic regression number of obs.	=	8096.000
LR chi2(26)	=	789.350
Prob > chi2	=	0.000
Log likelihood	=	-3370.977
Pseudo R2	=	0.105

Modern contraceptive use	Coef.	Std. Err.	z	P>z	95% Conf. interval	
Constant	-0.184	0.240	-0.760	0.445	-0.655	0.287
Age	0.002	0.008	0.240	0.810	-0.014	0.017
Children ever born	0.054	0.025	2.180	0.029	0.005	0.103

2^0 / 30 education (ref.)						
No education	-1.151	0.108	-10.650	0.000	-1.363	-0.939
1^0 education	-0.489	0.114	-4.280	0.000	-0.713	-0.265
Urban(ref.)						
rural	-0.630	0.084	-7.470	0.000	-0.796	-0.465
Catholic / protestant (ref.)						
No religion	0.154	0.360	0.430	0.669	-0.552	0.859
Islam	-0.283	0.072	-3.920	0.000	-0.424	-0.141
Traditional / Other	-0.997	0.198	-5.030	0.000	-1.386	-0.608
No partner	-0.829	0.177	-4.690	0.000	-1.175	-0.483
Complication information	-0.217	0.065	-3.330	0.001	-0.344	-0.089
Health facility permit	0.131	0.097	1.350	0.178	-0.060	0.322
Health facility money	-0.181	0.072	-2.510	0.012	-0.323	-0.039
Health facility distance	-0.160	0.077	-2.080	0.037	-0.310	-0.010
Health facility alone	-0.204	0.107	-1.910	0.056	-0.413	0.005
BoucledeMo~n (ref)						
Cascades	0.505	0.170	2.970	0.003	0.171	0.839
Centre	0.437	0.136	3.200	0.001	0.170	0.704
CentreEst	-0.287	0.162	-1.770	0.077	-0.606	0.031
CentreNord	-0.129	0.163	-0.790	0.429	-0.448	0.191
CentreOuest	-0.395	0.167	-2.360	0.018	-0.722	-0.068
CentreSud	0.525	0.168	3.120	0.002	0.195	0.855
Est	0.354	0.152	2.330	0.020	0.056	0.651
HautsBasins	0.954	0.126	7.570	0.000	0.707	1.201
Nord	0.112	0.158	0.710	0.480	-0.198	0.421
Plateau-Central	0.069	0.181	0.380	0.703	-0.287	0.425
Sahel	0.098	0.184	0.530	0.594	-0.263	0.459
SudOuest	0.393	0.230	1.710	0.087	-0.058	0.844

Adequate antenatal visits

Logistic regression number of obs.	=	8089.000
LR chi2(26)	=	531.960
Prob > chi2	=	0.000
Log likelihood	=	-5081.497
Pseudo R2	=	0.050

Adequate antenatal visits	Coef.	Std. Err.	z	P>z	95% Conf. interval	
Constant	0.001	0.192	0.000	0.998	-0.376	0.377
Age	0.013	0.006	2.070	0.039	0.001	0.025
Children ever born	-0.066	0.019	-3.390	0.001	-0.104	-0.028
2^0 / 30 education (ref.)						
No education	-0.626	0.099	-6.330	0.000	-0.820	-0.432
1^0 education	-0.435	0.107	-4.060	0.000	-0.646	-0.225
Urban(ref.)						
rural	-0.053	0.072	-0.740	0.462	-0.194	0.088
Catholic / protestant (ref.)						
No religion	-0.111	0.290	-0.380	0.702	-0.678	0.457
Islam	-0.150	0.057	-2.620	0.009	-0.262	-0.038
Traditional / Other	-0.399	0.123	-3.230	0.001	-0.640	-0.157
No partner	-0.239	0.133	-1.790	0.073	-0.500	0.023
No complication information	0.004	0.050	0.090	0.928	-0.093	0.102
Health facility permit	0.237	0.073	3.260	0.001	0.094	0.379
Health facility money	-0.075	0.058	-1.280	0.199	-0.189	0.039
Health facility distance	-0.193	0.057	-3.390	0.001	-0.305	-0.081
Health facility alone	0.095	0.078	1.200	0.228	-0.059	0.248
BoucledeMo~n (ref)						
Cascades	-0.062	0.135	-0.460	0.646	-0.326	0.202
Centre	0.523	0.108	4.830	0.000	0.311	0.736
CentreEst	0.893	0.104	8.580	0.000	0.689	1.097
CentreNord	-0.091	0.110	-0.830	0.408	-0.308	0.125

CentreOuest	-0.083	0.112	-0.740	0.458	-0.302	0.136
CentreSud	0.594	0.125	4.740	0.000	0.348	0.839
Est	0.043	0.113	0.380	0.702	-0.178	0.264
HautsBasins	-0.165	0.101	-1.640	0.102	-0.363	0.033
Nord	-0.171	0.113	-1.510	0.130	-0.392	0.050
Plateau-Central	0.330	0.123	2.690	0.007	0.090	0.571
Sahel	-0.734	0.145	-5.060	0.000	-1.018	-0.450
SudOuest	0.521	0.161	3.250	0.001	0.206	0.836

Facility-based childbirth

Logistic regression number of obs.	=	8094.000
LR chi2(26)	=	1429.880
Prob > chi2	=	0.000
Log likelihood	=	-3410.736
Pseudo R2	=	0.173

Facility-based childbirth	Coef.	Std. Err.	z	P>z	95% Conf. interval	
Constant	4.835	0.367	13.180	0.000	4.116	5.555
Age	-0.001	0.008	-0.170	0.863	-0.017	0.014
Children ever born	-0.057	0.024	-2.420	0.016	-0.104	-0.011
2^0 / 30 education (ref.)						
No education	-1.376	0.286	-4.820	0.000	-1.936	-0.816
1^0 education	-0.620	0.304	-2.040	0.042	-1.217	-0.023
Urban(ref.)						
rural	-1.318	0.141	-9.350	0.000	-1.594	-1.042
Catholic / protestant (ref.)						
No religion	0.391	0.381	1.030	0.305	-0.356	1.137
Islam	-0.326	0.081	-4.030	0.000	-0.484	-0.167
Traditional / Other	-0.741	0.129	-5.730	0.000	-0.995	-0.488
No partner	0.032	0.237	0.140	0.892	-0.432	0.496
No complication information	-0.441	0.063	-7.030	0.000	-0.565	-0.318
Health facility permit	0.386	0.099	3.890	0.000	0.191	0.580
Health facility money	-0.012	0.075	-0.150	0.877	-0.159	0.135
Health facility distance	-0.937	0.068	-13.820	0.000	-1.070	-0.804
Health facility alone	0.064	0.102	0.630	0.529	-0.135	0.263
BoucledeMo~n (ref)						
Cascades	0.406	0.173	2.350	0.019	0.067	0.744
Centre	1.659	0.270	6.140	0.000	1.130	2.189
CentreEst	0.721	0.143	5.030	0.000	0.440	1.002
CentreNord	0.005	0.126	0.040	0.967	-0.242	0.252
CentreOuest	0.000	0.000	-0.590	0.553	-0.001	0.000
CentreSud	1.009	0.195	5.170	0.000	0.626	1.392
Est	-0.351	0.125	-2.810	0.005	-0.595	-0.106
HautsBasins	0.013	0.124	0.100	0.919	-0.230	0.255
Nord	-0.139	0.123	-1.130	0.260	-0.380	0.103
Plateau-Central	0.638	0.163	3.920	0.000	0.319	0.957
Sahel	-0.833	0.132	-6.300	0.000	-1.092	-0.574
SudOuest	-0.455	0.182	-2.500	0.012	-0.812	-0.099

C-section

Logistic regression number of obs.	=	8040.000
LR chi2(25)	=	227.880
Prob > chi2	=	0.000
Log likelihood	=	-878.627
Pseudo R2	=	0.115

C-section	Coef.	Std. Err.	z	P>z	95% Conf. interval	
Constant						
Age	0.046	0.017	2.740	0.006	0.013	0.079
Children ever born	-0.127	0.058	-2.180	0.030	-0.242	-0.013
2^0 / 30 education (ref.)						
No education	-0.376	0.215	-1.750	0.080	-0.797	0.045
1^0 education	-0.118	0.222	-0.530	0.596	-0.553	0.318
Urban(ref.)						
rural	-0.996	0.198	-5.040	0.000	-1.383	-0.608
Catholic / protestant (ref.)	-0.129	0.156	-0.830	0.409	-0.436	0.178
No religion	-0.046	0.445	-0.100	0.918	-0.917	0.826
Islam	-0.778	0.395	-1.970	0.049	-1.552	-0.003
Traditional / Other	-0.409	0.152	-2.680	0.007	-0.708	-0.110
No partner	0.100	0.215	0.470	0.641	-0.322	0.522
No complication information	-0.147	0.165	-0.890	0.375	-0.471	0.177
Health facility permit	-0.166	0.184	-0.900	0.366	-0.527	0.194
Health facility money	0.115	0.222	0.520	0.604	-0.321	0.551
Health facility distance						
Health facility alone	-0.600	0.518	-1.160	0.247	-1.616	0.416
BoucledeMo~n (ref)						
Cascades	0.574	0.293	1.960	0.050	-0.001	1.150
Centre	-0.506	0.395	-1.280	0.200	-1.281	0.269
CentreEst	-1.354	0.593	-2.280	0.022	-2.516	-0.192
CentreNord	-0.150	0.371	-0.400	0.687	-0.877	0.578
CentreOuest	0.252	0.388	0.650	0.515	-0.508	1.012
CentreSud	-0.310	0.430	-0.720	0.472	-1.153	0.533
Est	0.080	0.309	0.260	0.795	-0.525	0.686
HautsBasins	-0.142	0.387	-0.370	0.713	-0.901	0.616
Nord	-0.017	0.426	-0.040	0.967	-0.852	0.817
Plateau-Central	-1.868	1.004	-1.860	0.063	-3.835	0.099
Sahel	-0.321	0.598	-0.540	0.591	-1.494	0.852
SudOuest	-3.217	0.524	-6.140	0.000	-4.244	-2.190

Logit Regressions for Probability of reproductive health care service use in Niger

Family planning exposure

Logistic regression number of obs.	=	6430.000
LR chi2(26)	=	1240.550
Prob > chi2	=	0.000
Log likelihood	=	-3832.964
Pseudo R2	=	0.139

Family planning exposure	Coef.	Std. Err.	z	P>z	95% Conf. interval	
_cons	1.432	0.226	6.330	0.000	0.989	1.876
Age	0.025	0.007	3.590	0.000	0.011	0.038
Children ever born	-0.001	0.018	-0.060	0.956	-0.036	0.034
2^0 / 30 education (ref.)						
No education	-0.979	0.143	-6.860	0.000	-1.258	-0.699
1^0 education	-0.348	0.160	-2.180	0.030	-0.661	-0.035
Urban(ref.)						
rural	-0.999	0.102	-9.760	0.000	-1.199	-0.798
Catholic / protestant (ref.)						
No religion						
Islam						
Traditional / Other						
No partner	-0.477	0.185	-2.580	0.010	-0.839	-0.115
No complication information	-0.842	0.060	-13.960	0.000	-0.960	-0.724
missing	-0.892	0.232	-3.850	0.000	-1.346	-0.437
don't know	-0.979	0.092	-10.600	0.000	-1.160	-0.798
Health facility permit	0.216	0.082	2.630	0.008	0.055	0.377
Health facility money	-0.083	0.066	-1.270	0.204	-0.212	0.045
Health facility distance	-0.238	0.070	-3.380	0.001	-0.376	-0.100

Health facility alone	0.362	0.075	4.840	0.000	0.215	0.508
Niamey (ref.)						
Agadez	-0.813	0.256	-3.170	0.002	-1.316	-0.311
Diffa	-1.320	0.235	-5.630	0.000	-1.780	-0.860
Dosso	-0.398	0.164	-2.430	0.015	-0.719	-0.077
Maradi	0.494	0.159	3.110	0.002	0.183	0.806
Tahoua	-0.261	0.155	-1.680	0.093	-0.565	0.043
Tillaberi	1.062	0.170	6.240	0.000	0.729	1.396
Zinder	-0.133	0.158	-0.840	0.403	-0.443	0.178

Modern contraceptive use

Logistic regression number of obs.	=	6437.000
LR chi2(26)	=	537.330
Prob > chi2	=	0.000
Log likelihood	=	-2515.194
Pseudo R2	=	0.097

Modern contraceptive use	Coef.	Std. Err.	z	P>z	95% Conf. interval	
_cons	0.714	0.254	2.810	0.005	0.216	1.213
Age	-0.026	0.009	-2.830	0.005	-0.043	-0.008
Children ever born	0.085	0.024	3.600	0.000	0.039	0.131
2^0 / 30 education (ref.)						
No education	-0.937	0.131	-7.140	0.000	-1.194	-0.679
1^0 education	-0.501	0.147	-3.400	0.001	-0.790	-0.212
Urban(ref.)						
rural	-0.928	0.110	-8.430	0.000	-1.144	-0.713
Catholic / protestant (ref.)						
No religion						
Islam						
Traditional / Other						
No partner	-1.796	0.358	-5.010	0.000	-2.499	-1.094
No complication information	-0.021	0.078	-0.270	0.785	-0.174	0.132
missing	0.475	0.297	1.600	0.110	-0.107	1.057
don't know	-0.715	0.162	-4.400	0.000	-1.034	-0.397
Health facility permit	-0.032	0.113	-0.280	0.779	-0.254	0.190
Health facility money	0.073	0.085	0.860	0.389	-0.093	0.240
Health facility distance	-0.164	0.094	-1.740	0.082	-0.349	0.021
Health facility alone	-0.174	0.105	-1.660	0.098	-0.381	0.032
Niamey (ref.)						
Agadez	-0.135	0.267	-0.500	0.614	-0.658	0.388
Diffa	-0.219	0.250	-0.880	0.380	-0.709	0.270
Dosso	0.111	0.161	0.690	0.489	-0.204	0.426
Maradi	-0.879	0.163	-5.400	0.000	-1.198	-0.560
Tahoua	-1.064	0.164	-6.490	0.000	-1.385	-0.743
Tillaberi	-0.237	0.169	-1.400	0.161	-0.568	0.095
Zinder	-0.197	0.154	-1.280	0.202	-0.499	0.105

Adequate antenatal visits

Logistic regression number of obs.	=	5640.000
LR chi2(26)	=	272.510
Prob > chi2	=	0.000
Log likelihood	=	-3652.991
Pseudo R2	=	0.036

Adequate antenatal visits	Coef.	Std. Err.	z	P>z	95% Conf. interval	
_cons	0.063	0.208	0.300	0.762	-0.344	0.470
Age	0.023	0.007	3.330	0.001	0.009	0.037
Children ever born	-0.047	0.018	-2.570	0.010	-0.082	-0.011
2^0 / 30 education (ref.)						
No education	-0.748	0.118	-6.310	0.000	-0.980	-0.515

1^0 education	-0.561	0.134	-4.190	0.000	-0.823	-0.299
Urban(ref.)						
rural	-0.154	0.093	-1.660	0.098	-0.337	0.028
Catholic / protestant (ref.)						
No religion						
Islam						
Traditional / Other						
No partner	0.128	0.181	0.710	0.479	-0.227	0.484
No complication information	-0.111	0.059	-1.890	0.059	-0.225	0.004
missing	-0.195	0.226	-0.860	0.389	-0.638	0.248
don't know						
Health facility permit	0.090	0.084	1.080	0.281	-0.074	0.254
Health facility money	-0.068	0.067	-1.010	0.312	-0.198	0.063
Health facility distance	-0.144	0.073	-1.970	0.049	-0.288	-0.001
Health facility alone	0.016	0.077	0.210	0.833	-0.135	0.167
Niamey (ref.)						
Agadez	-0.017	0.246	-0.070	0.946	-0.499	0.465
Diffa	-0.246	0.226	-1.090	0.276	-0.688	0.197
Dosso	-0.348	0.147	-2.360	0.018	-0.637	-0.059
Maradi	0.461	0.139	3.310	0.001	0.188	0.734
Tahoua	-0.234	0.139	-1.680	0.092	-0.506	0.038
Tillaberi	-0.601	0.152	-3.970	0.000	-0.899	-0.304
Zinder	0.262	0.140	1.870	0.062	-0.013	0.536

No information on pregnancy complication (Don't know) dropped and 767 obs not used

Facility-based childbirth

Logistic regression number of obs.	=	6435.000
LR chi2(26)	=	2077.330
Prob > chi2	=	0.000
Log likelihood	=	-3242.137
Pseudo R2	=	0.243

**Facility-based childbirth**	Coef.	Std. Err.	z	P>z	95% Conf. interval	
_cons	2.764	0.266	10.390	0.000	2.243	3.285
Age	0.024	0.008	3.090	0.002	0.009	0.039
Children ever born	-0.097	0.020	-4.820	0.000	-0.137	-0.058
2^0 / 30 education (ref.)						
No education	-1.057	0.154	-6.880	0.000	-1.358	-0.756
1^0 education	-0.269	0.173	-1.550	0.121	-0.609	0.071
Urban(ref.)						
rural	-2.119	0.115	-18.500	0.000	-2.344	-1.895
Catholic / protestant (ref.)						
No religion						
Islam						
Traditional / Other						
No partner	0.509	0.218	2.330	0.020	0.081	0.936
No complication information	-0.419	0.065	-6.440	0.000	-0.547	-0.292
missing	-0.474	0.247	-1.920	0.055	-0.958	0.010
don't know	-2.512	0.177	-14.210	0.000	-2.858	-2.165
Health facility permit	0.249	0.094	2.660	0.008	0.065	0.432
Health facility money	0.028	0.072	0.390	0.694	-0.113	0.170
Health facility distance	-0.625	0.080	-7.840	0.000	-0.782	-0.469
Health facility alone	-0.038	0.085	-0.450	0.653	-0.206	0.129
Niamey (ref.)						
Agadez	0.411	0.353	1.170	0.244	-0.280	1.103
Diffa	0.327	0.262	1.250	0.212	-0.187	0.841
Dosso	-0.152	0.204	-0.750	0.455	-0.551	0.247
Maradi	-0.431	0.197	-2.190	0.028	-0.817	-0.046
Tahoua	-0.290	0.196	-1.480	0.140	-0.674	0.095
Tillaberi	-0.068	0.204	-0.330	0.740	-0.469	0.333
Zinder	-0.705	0.198	-3.560	0.000	-1.093	-0.316

C-section

Logistic regression number of obs.	=	6437.000
LR chi2(26)	=	143.180
Prob > chi2	=	0.000
Log likelihood	=	-545.454
Pseudo R2	=	0.116

**C-section**	Coef.	Std. Err.	z	P>z	95% Conf. interval	
_cons	-2.266	0.552	-4.100	0.000	-3.349	-1.184
Age	0.028	0.021	1.340	0.182	-0.013	0.068
Children ever born	-0.034	0.058	-0.590	0.558	-0.149	0.080
2^0 / 30 education (ref.)						
No education	-0.812	0.268	-3.030	0.002	-1.337	-0.287
1^0 education	-0.535	0.299	-1.790	0.074	-1.122	0.051
Urban(ref.)						
rural	-0.766	0.284	-2.700	0.007	-1.322	-0.210
Catholic / protestant (ref.)						
No religion						
Islam						
Traditional / Other						
No partner	0.068	0.452	0.150	0.881	-0.818	0.953
No complication information	-0.087	0.202	-0.430	0.668	-0.484	0.310
missing	0.813	0.535	1.520	0.128	-0.235	1.861
don't know	-0.692	0.507	-1.370	0.172	-1.686	0.301
Health facility permit	0.469	0.278	1.690	0.092	-0.076	1.014
Health facility money	-0.556	0.231	-2.400	0.016	-1.009	-0.102
Health facility distance	-0.032	0.249	-0.130	0.899	-0.520	0.457
Health facility alone	-0.273	0.274	-0.990	0.320	-0.810	0.265
Niamey (ref.)						
Agadez	-0.774	0.598	-1.300	0.195	-1.945	0.397
Diffa	-1.838	1.038	-1.770	0.077	-3.873	0.196
Dosso	-1.033	0.399	-2.590	0.010	-1.815	-0.250
Maradi	-1.117	0.373	-2.990	0.003	-1.849	-0.385
Tahoua	-0.765	0.329	-2.330	0.020	-1.410	-0.121
Tillaberi	-0.821	0.397	-2.070	0.038	-1.599	-0.044
Zinder	-1.416	0.399	-3.540	0.000	-2.199	-0.633

Logit Regressions for Probability of reproductive health care service use in Nigeria

Family planning exposure

Logistic regression number of obs.	=	15541.000
LR chi2(21)	=	4043.480
Prob > chi2	=	0.000
Log likelihood	=	-8429.620
Pseudo R2	=	0.193

Family planning exposure	Coef.	Std. Err.	z	P>z	95% Conf. interval	
_cons	0.768	0.115	6.690	0.000	0.543	0.994
Age	0.028	0.004	6.800	0.000	0.020	0.035
Children ever born	-0.023	0.012	-1.910	0.056	-0.046	0.001
2^0 / 30 education (ref.)						
No education	-1.269	0.059	-21.410	0.000	-1.385	-1.153
1^0 education	-0.607	0.050	-12.070	0.000	-0.706	-0.509
Urban(ref.)						
rural	-0.610	0.041	-14.800	0.000	-0.691	-0.529
Catholic / protestant (ref.)						
Islam	0.233	0.056	4.140	0.000	0.123	0.343
Traditional / Other	-1.161	0.286	-4.050	0.000	-1.722	-0.599
No partner	-0.201	0.078	-2.600	0.009	-0.353	-0.049
No complication information	-0.725	0.048	-14.970	0.000	-0.819	-0.630
Don't know	-0.679	0.172	-3.950	0.000	-1.016	-0.343

missing	-0.950	0.058	-16.250	0.000	-1.064	-0.835
Health facility permit	-0.055	0.076	-0.730	0.466	-0.204	0.094
Health facility money	-0.154	0.045	-3.410	0.001	-0.243	-0.066
Health facility distance	-0.035	0.054	-0.650	0.517	-0.140	0.070
Health facility alone	0.069	0.076	0.900	0.366	-0.080	0.218
Health facility attitude	0.306	0.062	4.940	0.000	0.184	0.427
South West (ref.)						
North Central	-1.282	0.068	-18.800	0.000	-1.415	-1.148
North East	-1.267	0.079	-16.090	0.000	-1.421	-1.113
North West	-0.676	0.068	-9.930	0.000	-0.809	-0.542
South East	-1.052	0.073	-14.430	0.000	-1.195	-0.910
South South	-0.779	0.070	-11.080	0.000	-0.917	-0.641

Modern contraceptive use

Logistic regression number of obs.	=	15590
LR chi2(21)	=	1738.580
Prob > chi2	=	0.000
Log likelihood	=	-5164.410
Pseudo R2	=	0.144

Modern contraceptive use	Coef.	Std. Err.	z	P>z	95% Conf. interval	
_cons	-1.075	0.149	-7.210	0.000	-1.367	-0.783
Age	0.008	0.005	1.490	0.137	-0.003	0.018
Children ever born	0.097	0.016	5.930	0.000	0.065	0.130
2^0 / 30 education (ref.)						
No education	-1.529	0.104	-14.680	0.000	-1.734	-1.325
1^0 education	-0.346	0.064	-5.380	0.000	-0.472	-0.220
Urban(ref.)						
rural	-0.380	0.059	-6.460	0.000	-0.496	-0.265
Catholic / protestant (ref.)						
Islam	-0.654	0.067	-9.700	0.000	-0.786	-0.522
Traditional / Other	-0.655	0.361	-1.810	0.070	-1.362	0.053
No partner	0.035	0.100	0.350	0.728	-0.161	0.231
No complication information	-0.099	0.068	-1.460	0.144	-0.231	0.034
Don't know	-0.688	0.298	-2.310	0.021	-1.273	-0.103
missing	-0.702	0.101	-6.930	0.000	-0.900	-0.503
Health facility permit	-0.415	0.135	-3.070	0.002	-0.680	-0.150
Health facility money	0.036	0.061	0.590	0.557	-0.083	0.155
Health facility distance	-0.139	0.078	-1.770	0.077	-0.293	0.015
Health facility alone	-0.228	0.121	-1.880	0.060	-0.466	0.009
Health facility attitude	0.193	0.085	2.260	0.024	0.026	0.360
South West (ref.)						
North Central	-0.160	0.079	-2.020	0.044	-0.315	-0.004
North East	-1.201	0.129	-9.310	0.000	-1.454	-0.948
North West	-0.615	0.090	-6.820	0.000	-0.792	-0.438
South East	-1.125	0.092	-12.240	0.000	-1.305	-0.945
South South	-0.412	0.080	-5.160	0.000	-0.568	-0.255

Adequate antenatal visits

Logistic regression number of obs.	=	11433.000
LR chi2(20)	=	1261.170
Prob > chi2	=	0.000
Log likelihood	=	-4550.815
Pseudo R2	=	0.122

Adequate antenatal visits	Coef.	Std. Err.	z	P>z	95% Conf. interval	
_cons	2.567	0.183	14.030	0.000	2.208	2.925
Age	0.039	0.006	6.460	0.000	0.027	0.051
Children ever born	-0.081	0.017	-4.700	0.000	-0.114	-0.047
2^0 / 30 education (ref.)						
No education	-0.554	0.077	-7.160	0.000	-0.705	-0.402

1^0 education	-0.434	0.074	-5.880	0.000	-0.578	-0.289
Urban(ref.)						
rural	-0.153	0.059	-2.610	0.009	-0.269	-0.038
Catholic / protestant (ref.)						
Islam	0.022	0.078	0.280	0.783	-0.132	0.175
Traditional / Other	0.090	0.357	0.250	0.802	-0.610	0.789
No partner	-0.054	0.116	-0.470	0.639	-0.281	0.172
No complication information	-0.438	0.056	-7.790	0.000	-0.548	-0.328
Don't know	-0.259	0.210	-1.230	0.218	-0.670	0.153
missing						
Health facility permit	-0.046	0.106	-0.440	0.663	-0.255	0.162
Health facility money	-0.211	0.063	-3.370	0.001	-0.334	-0.088
Health facility distance	-0.430	0.070	-6.170	0.000	-0.567	-0.294
Health facility alone	0.164	0.108	1.520	0.128	-0.047	0.376
Health facility attitude	0.059	0.088	0.670	0.503	-0.113	0.231
South West (ref.)						
North Central	-1.301	0.117	-11.160	0.000	-1.529	-1.072
North East	-1.585	0.121	-13.050	0.000	-1.823	-1.347
North West	-1.653	0.115	-14.380	0.000	-1.878	-1.428
South East	-0.407	0.141	-2.880	0.004	-0.684	-0.130
South South	-0.866	0.132	-6.550	0.000	-1.125	-0.607

No information on pregnancy complication (missing) dropped and 3681 obs not used

Facility-based childbirth

Logistic regression number of obs.	=	15569.000
LR chi2(26)	=	7299.610
Prob > chi2	=	0.000
Log likelihood	=	-7112.824
Pseudo R2	=	0.339

Facility-based childbirth	Coef.	Std. Err.	z	P>z	95% Conf. interval	
_cons	1.416	0.128	11.090	0.000	1.166	1.667
Age	0.040	0.005	8.810	0.000	0.031	0.049
Children ever born	-0.137	0.014	-10.060	0.000	-0.164	-0.110
2^0 / 30 education (ref.)						
No education	-0.999	0.060	-16.550	0.000	-1.117	-0.881
1^0 education	-0.586	0.053	-10.990	0.000	-0.691	-0.482
Urban(ref.)						
rural	-0.576	0.046	-12.600	0.000	-0.665	-0.486
Catholic / protestant (ref.)						
Islam	-0.436	0.057	-7.640	0.000	-0.548	-0.324
Traditional / Other	-0.358	0.237	-1.510	0.131	-0.823	0.106
No partner	-0.243	0.084	-2.900	0.004	-0.408	-0.079
No complication information	-0.402	0.049	-8.240	0.000	-0.498	-0.306
Don't know	-0.625	0.169	-3.710	0.000	-0.956	-0.295
missing	-2.219	0.076	-29.050	0.000	-2.369	-2.069
Health facility permit	-0.062	0.091	-0.690	0.492	-0.240	0.115
Health facility money	-0.239	0.049	-4.850	0.000	-0.336	-0.143
Health facility distance	-0.244	0.059	-4.160	0.000	-0.360	-0.129
Health facility alone	-0.079	0.088	-0.900	0.367	-0.252	0.093
Health facility attitude	0.198	0.070	2.820	0.005	0.060	0.336
South West (ref.)						
North Central	-0.157	0.072	-2.180	0.030	-0.299	-0.016
North East	-0.868	0.080	-10.880	0.000	-1.024	-0.711
North West	-1.330	0.072	-18.460	0.000	-1.471	-1.189
South East	0.307	0.090	3.430	0.001	0.132	0.483
South South	-0.800	0.077	-10.450	0.000	-0.950	-0.650

C-section

Logistic regression number of obs.	=	15590.000

LR chi2(26)	=	524.010
Prob > chi2	=	0.000
Log likelihood	=	-1798.503
Pseudo R2	=	0.127

C-section	Coef.	Std. Err.	z	P>z	95% Conf. interval	
_cons	-4.090	0.279	-14.650	0.000	-4.637	-3.543
Age	0.080	0.009	8.510	0.000	0.062	0.099
Children ever born	-0.294	0.035	-8.420	0.000	-0.363	-0.226
2^0 / 30 education (ref.)						
No education	-0.669	0.206	-3.250	0.001	-1.072	-0.265
1^0 education	-0.343	0.142	-2.410	0.016	-0.621	-0.064
Urban(ref.)						
rural	-0.537	0.118	-4.560	0.000	-0.767	-0.306
Catholic / protestant (ref.)						
Islam	-0.485	0.139	-3.480	0.001	-0.758	-0.211
Traditional / Other	0.607	0.514	1.180	0.237	-0.399	1.614
No partner	-0.028	0.190	-0.150	0.882	-0.401	0.345
No complication information	-0.606	0.149	-4.060	0.000	-0.899	-0.313
Don't know	-2.058	1.159	-1.780	0.076	-4.331	0.214
missing	-0.992	0.248	-4.010	0.000	-1.478	-0.507
Health facility permit	0.082	0.257	0.320	0.749	-0.421	0.585
Health facility money	-0.320	0.125	-2.560	0.011	-0.566	-0.075
Health facility distance	-0.046	0.164	-0.280	0.780	-0.368	0.276
Health facility alone	-0.252	0.257	-0.980	0.328	-0.755	0.252
Health facility attitude	0.148	0.167	0.880	0.376	-0.180	0.476
South West (ref.)						
North Central	0.270	0.156	1.720	0.085	-0.037	0.576
North East	0.304	0.214	1.420	0.156	-0.116	0.723
North West	-0.346	0.206	-1.680	0.093	-0.750	0.058
South East	0.093	0.157	0.590	0.555	-0.215	0.400
South South	0.324	0.150	2.160	0.030	0.031	0.617

Logit Regressions for Probability of reproductive health care service use in Ghana

Family planning exposure

Logistic regression number of obs.	=	3200
LR chi2(26)	=	304.590
Prob > chi2	=	0.000
Log likelihood	=	-1700.016
Pseudo R2	=	0.082

Family planning exposure	Coef.	Std. Err.	z	P>z	95% Conf. interval	
cons	0.908	0.251	3.620	0.000	0.417	1.400
Age	0.022	0.009	2.490	0.013	0.005	0.039
Children ever born	-0.060	0.033	-1.830	0.067	-0.125	0.004
2^0 / 30 education (ref.)						
No education	-0.736	0.128	-5.740	0.000	-0.988	-0.485
1^0 education	-0.446	0.112	-3.970	0.000	-0.666	-0.226
Urban(ref.)						
rural	-0.035	0.095	-0.370	0.711	-0.220	0.150
Catholic / protestant (ref.)						
Islam	0.038	0.138	0.270	0.785	-0.233	0.308
Traditional / Other	-0.132	0.218	-0.610	0.544	-0.559	0.295
No partner	-0.202	0.113	-1.790	0.074	-0.424	0.020
No complication information	-0.221	0.116	-1.910	0.056	-0.448	0.006
Health facility permit	0.067	0.190	0.350	0.724	-0.305	0.440
Health facility money	-0.332	0.099	-3.360	0.001	-0.525	-0.138
Health facility distance	-0.107	0.120	-0.890	0.372	-0.342	0.128
Health facility alone	0.023	0.145	0.160	0.875	-0.261	0.307
Uninsured	-0.238	0.093	-2.560	0.010	-0.419	-0.056
Ashanti (ref.)						

Western	0.814	0.165	4.930	0.000	0.490	1.137
Central	0.781	0.160	4.880	0.000	0.467	1.095
Volta	0.517	0.188	2.740	0.006	0.148	0.887
Eastern	-0.348	0.148	-2.360	0.018	-0.638	-0.059
Greater Accra	0.559	0.143	3.900	0.000	0.278	0.840
Brong Ahafo	-0.795	0.154	-5.150	0.000	-1.097	-0.493
Northern	-0.017	0.235	-0.070	0.943	-0.478	0.445
Upper East	-0.969	0.343	-2.830	0.005	-1.641	-0.297
Upper West	0.048	0.353	0.140	0.892	-0.644	0.740

Modern contraceptive use

Logistic regression number of obs.	=	3200
LR chi2(26)	=	105.220
Prob > chi2	=	0.000
Log likelihood	=	-1798.573
Pseudo R2	=	0.028

Modern contraceptive use	Coef.	Std. Err.	z	P>z	95% Conf. interval	
cons	-0.756	0.249	-3.030	0.002	-1.245	-0.267
Age	-0.033	0.009	-3.800	0.000	-0.050	-0.016
Children ever born	0.145	0.032	4.550	0.000	0.082	0.207
2^0 / 30 education (ref.)						
No education	-0.310	0.136	-2.280	0.023	-0.576	-0.044
1^0 education	-0.006	0.110	-0.060	0.954	-0.222	0.209
Urban(ref.)						
rural	0.132	0.092	1.430	0.152	-0.048	0.312
Catholic / protestant (ref.)						
Islam	-0.143	0.144	-0.990	0.321	-0.424	0.139
Traditional / Other	0.135	0.215	0.620	0.532	-0.288	0.557
No partner	-0.069	0.112	-0.620	0.535	-0.289	0.150
No complication information	0.081	0.117	0.690	0.489	-0.148	0.310
Health facility permit	0.065	0.192	0.340	0.735	-0.311	0.441
Health facility money	-0.090	0.098	-0.910	0.361	-0.282	0.103
Health facility distance	-0.245	0.124	-1.980	0.048	-0.487	-0.002
Health facility alone	0.091	0.149	0.610	0.539	-0.200	0.383
Uninsured	0.244	0.090	2.720	0.006	0.068	0.419
Ashanti (ref.)						
Western	0.288	0.151	1.920	0.055	-0.007	0.583
Central	0.486	0.144	3.380	0.001	0.204	0.768
Volta	0.665	0.174	3.830	0.000	0.324	1.005
Eastern	0.312	0.158	1.970	0.049	0.002	0.623
Greater Accra	0.137	0.140	0.980	0.325	-0.136	0.411
Brong Ahafo	0.636	0.162	3.930	0.000	0.319	0.953
Northern	-0.605	0.331	-1.830	0.067	-1.253	0.043
Upper East	0.798	0.358	2.230	0.026	0.095	1.500
Upper West	0.533	0.376	1.420	0.156	-0.204	1.269

Adequate antenatal visits

Logistic regression number of obs.	=	3189
LR chi2(26)	=	215.170
Prob > chi2	=	0.000
Log likelihood	=	-758.868
Pseudo R2	=	0.124

Adequate antenatal visits	Coef.	Std. Err.	z	P>z	95% Conf. interval	
cons	2.147	0.453	4.740	0.000	1.260	3.034
Age	0.098	0.016	6.180	0.000	0.067	0.130
Children ever born	-0.258	0.055	-4.660	0.000	-0.366	-0.149

2^0 / 30 education (ref.)						
No education	-0.509	0.207	-2.460	0.014	-0.914	-0.103
1^0 education	-0.288	0.177	-1.630	0.103	-0.635	0.059
Urban(ref.)						
rural	-0.383	0.158	-2.420	0.015	-0.694	-0.073
Catholic / protestant (ref.)						
Islam	0.239	0.257	0.930	0.352	-0.265	0.743
Traditional / Other	-0.248	0.297	-0.840	0.404	-0.830	0.334
No partner	-0.081	0.180	-0.450	0.652	-0.433	0.271
No complication information	-0.696	0.172	-4.050	0.000	-1.033	-0.359
Health facility permit	0.210	0.316	0.660	0.507	-0.410	0.829
Health facility money	-0.272	0.161	-1.690	0.090	-0.588	0.043
Health facility distance	0.202	0.192	1.060	0.291	-0.173	0.578
Health facility alone	-0.647	0.211	-3.060	0.002	-1.061	-0.233
Uninsured	-0.596	0.146	-4.070	0.000	-0.882	-0.309
Ashanti (ref.)						
Western	-0.529	0.299	-1.770	0.077	-1.115	0.056
Central	-0.552	0.290	-1.900	0.057	-1.121	0.018
Volta	-1.455	0.294	-4.950	0.000	-2.032	-0.879
Eastern	-1.662	0.258	-6.440	0.000	-2.168	-1.156
Greater Accra	-1.122	0.281	-4.000	0.000	-1.672	-0.572
Brong Ahafo	-0.659	0.325	-2.030	0.043	-1.296	-0.022
Northern	-1.642	0.390	-4.210	0.000	-2.407	-0.877
Upper East	0.452	1.300	0.350	0.728	-2.095	3.000
Upper West	-0.522	0.691	-0.760	0.450	-1.875	0.831

Facility-based childbirth

Logistic regression number of obs.	=	3199
LR chi2(26)	=	532.830
Prob > chi2	=	0.000
Log likelihood	=	-1174.789
Pseudo R2	=	0.185

Facility-based childbirth	Coef.	Std. Err.	z	P>z	95% Conf. interval	
cons	2.434	0.329	7.390	0.000	1.789	3.080
Age	0.062	0.012	5.360	0.000	0.039	0.084
Children ever born	-0.275	0.041	-6.790	0.000	-0.354	-0.196
2^0 / 30 education (ref.)						
No education	-0.746	0.151	-4.930	0.000	-1.043	-0.449
1^0 education	-0.564	0.133	-4.240	0.000	-0.825	-0.303
Urban(ref.)						
rural	-1.279	0.124	-10.280	0.000	-1.523	-1.035
Catholic / protestant (ref.)						
Islam	0.168	0.187	0.890	0.371	-0.199	0.534
Traditional / Other	-0.186	0.245	-0.760	0.447	-0.666	0.293
No partner	-0.024	0.142	-0.170	0.864	-0.302	0.254
No complication information	-0.490	0.140	-3.510	0.000	-0.763	-0.216
Health facility permit	-0.168	0.230	-0.730	0.464	-0.618	0.282
Health facility money	0.190	0.124	1.530	0.125	-0.053	0.433
Health facility distance	-0.233	0.143	-1.630	0.103	-0.514	0.047
Health facility alone	-0.257	0.177	-1.450	0.147	-0.605	0.091
Uninsured	-0.399	0.113	-3.540	0.000	-0.620	-0.178
Ashanti (ref.)						
Western	-0.647	0.190	-3.400	0.001	-1.020	-0.273
Central	-0.866	0.182	-4.760	0.000	-1.223	-0.509
Volta	-0.728	0.225	-3.230	0.001	-1.169	-0.286
Eastern	-0.834	0.195	-4.270	0.000	-1.216	-0.452
Greater Accra	-0.154	0.230	-0.670	0.505	-0.605	0.298
Brong Ahafo	-0.116	0.237	-0.490	0.624	-0.580	0.348
Northern	-1.674	0.284	-5.900	0.000	-2.230	-1.118
Upper East	0.517	0.731	0.710	0.479	-0.916	1.950

Upper West	-0.020	0.471	-0.040	0.967	-0.943	0.904

C-section

Logistic regression number of obs.	=	3200
LR chi2(26)	=	270.920
Prob > chi2	=	0.000
Log likelihood	=	-1281.001
Pseudo R2	=	0.096

C-section	Coef.	Std. Err.	z	P>z	95% Conf. interval	
cons	-3.748	0.309	-12.130	0.000	-4.353	-3.142
Age	0.117	0.010	11.320	0.000	0.097	0.138
Children ever born	-0.362	0.042	-8.610	0.000	-0.444	-0.280
2^0 / 30 education (ref.)						
No education	-0.597	0.189	-3.170	0.002	-0.967	-0.228
1^0 education	-0.128	0.148	-0.870	0.386	-0.419	0.162
Urban(ref.)						
rural	-0.341	0.120	-2.830	0.005	-0.577	-0.105
Catholic / protestant (ref.)						
Islam	0.312	0.166	1.890	0.059	-0.012	0.637
Traditional / Other	0.516	0.284	1.820	0.069	-0.040	1.072
No partner	-0.052	0.144	-0.360	0.719	-0.335	0.231
No complication information	-0.423	0.170	-2.490	0.013	-0.755	-0.090
Health facility permit	-0.765	0.293	-2.610	0.009	-1.340	-0.190
Health facility money	-0.087	0.125	-0.690	0.488	-0.333	0.159
Health facility distance	0.118	0.157	0.750	0.451	-0.189	0.426
Health facility alone	0.012	0.184	0.060	0.948	-0.348	0.372
Uninsured	-0.112	0.115	-0.980	0.329	-0.337	0.113
Ashanti (ref.)						
Western	-0.070	0.183	-0.380	0.701	-0.430	0.289
Central	0.011	0.178	0.060	0.949	-0.337	0.360
Volta	-0.749	0.256	-2.930	0.003	-1.251	-0.248
Eastern	-0.440	0.210	-2.090	0.037	-0.852	-0.027
Greater Accra	-0.175	0.150	-1.170	0.241	-0.469	0.118
Brong Ahafo	-0.479	0.220	-2.180	0.029	-0.909	-0.048
Northern	-1.110	0.400	-2.780	0.005	-1.893	-0.327
Upper East	-0.764	0.534	-1.430	0.153	-1.811	0.283
Upper West	-0.428	0.551	-0.780	0.437	-1.508	0.652

Logit Regressions for Probability of reproductive health care service use in Senegal

Family planning exposure

Logistic regression number of obs.	=	6559
LR chi2(26)	=	546.710
Prob > chi2	=	0.000
Log likelihood	=	-4252.716
Pseudo R2	=	0.060

Family planning exposure	Coef.	Std. Err.	z	P>z	95% Conf. interval	
cons	0.673	0.220	3.050	0.002	0.241	1.105
Age	0.026	0.006	4.720	0.000	0.015	0.037
Children ever born	-0.059	0.018	-3.270	0.001	-0.094	-0.024
2^0 / 30 education (ref.)						
No education	-0.670	0.080	-8.380	0.000	-0.827	-0.513
1^0 education	-0.239	0.083	-2.870	0.004	-0.403	-0.076
Urban(ref.)						
rural	-0.218	0.064	-3.380	0.001	-0.344	-0.092
Catholic / protestant (ref.)						
Islam	0.064	0.153	0.420	0.678	-0.237	0.364

Traditional / Other	-2.793	1.364	-2.050	0.041	-5.466	-0.119
No partner	-0.353	0.103	-3.430	0.001	-0.555	-0.151
No complication information	-0.317	0.056	-5.680	0.000	-0.427	-0.208
Dakar (ref.)						
Ziguinchor	-0.900	0.156	-5.750	0.000	-1.206	-0.593
Diourbel	-0.511	0.098	-5.200	0.000	-0.703	-0.318
SaintLouis	-0.875	0.121	-7.250	0.000	-1.112	-0.639
Tambacounda	-0.798	0.172	-4.650	0.000	-1.135	-0.462
Kaolack	-0.504	0.113	-4.470	0.000	-0.725	-0.283
This	-0.013	0.090	-0.150	0.881	-0.190	0.163
Louga	-0.789	0.123	-6.410	0.000	-1.030	-0.548
Fatick	-0.612	0.134	-4.570	0.000	-0.875	-0.350
Kolda	-0.640	0.168	-3.810	0.000	-0.968	-0.311
Matam	-1.214	0.158	-7.660	0.000	-1.525	-0.904
Kaffrine	-0.518	0.183	-2.830	0.005	-0.877	-0.159
Kedougou	-1.044	0.280	-3.720	0.000	-1.593	-0.494
Sedhiou	-0.702	0.174	-4.050	0.000	-1.042	-0.362

Modern contraceptive use

Logistic regression number of obs.	=	6559
LR chi2(26)	=	539.000
Prob > chi2	=	0.000
Log likelihood	=	-3750.525
Pseudo R2	=	0.067

Family planning exposure	Coef.	Std. Err.	z	P>z	95% Conf. interval	
cons	0.282	0.229	1.230	0.218	-0.167	0.731
Age	-0.005	0.006	-0.920	0.358	-0.017	0.006
Children ever born	0.126	0.019	6.520	0.000	0.088	0.164
2^0 / 30 education (ref.)						
No education	-0.660	0.082	-8.020	0.000	-0.821	-0.499
1^0 education	-0.105	0.082	-1.280	0.202	-0.265	0.056
Urban(ref.)						
rural	-0.424	0.071	-6.020	0.000	-0.562	-0.286
Catholic / protestant (ref.)						
Islam	-0.407	0.153	-2.670	0.008	-0.706	-0.108
Traditional / Other	-0.074	0.641	-0.120	0.908	-1.331	1.183
No partner	-0.494	0.115	-4.300	0.000	-0.719	-0.269
No complication information	-0.152	0.060	-2.530	0.011	-0.269	-0.034
Dakar (ref.)						
Ziguinchor	-0.536	0.168	-3.200	0.001	-0.865	-0.208
Diourbel	-0.960	0.116	-8.270	0.000	-1.188	-0.733
SaintLouis	0.007	0.123	0.060	0.956	-0.233	0.247
Tambacounda	-0.804	0.203	-3.960	0.000	-1.201	-0.406
Kaolack	-0.524	0.122	-4.280	0.000	-0.764	-0.284
This	-0.009	0.089	-0.110	0.915	-0.184	0.165
Louga	-0.123	0.130	-0.940	0.346	-0.378	0.132
Fatick	-0.346	0.144	-2.400	0.017	-0.629	-0.063
Kolda	-0.812	0.197	-4.110	0.000	-1.199	-0.425
Matam	-0.983	0.194	-5.060	0.000	-1.363	-0.602
Kaffrine	-0.654	0.218	-3.000	0.003	-1.081	-0.227
Kedougou	-0.801	0.330	-2.430	0.015	-1.448	-0.154
Sedhiou	-0.862	0.210	-4.110	0.000	-1.273	-0.451

Adequate antenatal visits

Logistic regression number of obs.	=	6390
LR chi2(26)	=	501.040
Prob > chi2	=	0.000
Log likelihood	=	-4106.388
Pseudo R2	=	0.058

Adequate antenatal visits	Coef.	Std. Err.	z	P>z	95% Conf. interval	
cons	0.675	0.230	2.940	0.003	0.225	1.126
Age	0.049	0.006	8.470	0.000	0.037	0.060
Children ever born	-0.197	0.019	-10.550	0.000	-0.234	-0.160
2^0 / 30 education (ref.)						
No education	-0.497	0.083	-6.000	0.000	-0.659	-0.335
1^0 education	-0.281	0.086	-3.250	0.001	-0.450	-0.111
Urban(ref.)						
rural	-0.282	0.066	-4.290	0.000	-0.411	-0.153
Catholic / protestant (ref.)						
Islam	-0.170	0.163	-1.050	0.296	-0.489	0.149
Traditional / Other	-0.139	0.598	-0.230	0.816	-1.311	1.032
No partner	-0.509	0.105	-4.840	0.000	-0.715	-0.303
No complication information	-0.409	0.057	-7.200	0.000	-0.520	-0.298
Dakar (ref.)						
Ziguinchor	-0.065	0.166	-0.390	0.694	-0.392	0.261
Diourbel	-0.323	0.101	-3.190	0.001	-0.521	-0.125
SaintLouis	-0.363	0.123	-2.960	0.003	-0.604	-0.122
Tambacounda	0.013	0.177	0.070	0.944	-0.335	0.360
Kaolack	-0.160	0.117	-1.370	0.170	-0.389	0.068
This	-0.124	0.092	-1.350	0.177	-0.303	0.056
Louga	-0.550	0.125	-4.400	0.000	-0.795	-0.305
Fatick	-0.326	0.137	-2.380	0.017	-0.595	-0.057
Kolda	-0.731	0.170	-4.300	0.000	-1.063	-0.398
Matam	-0.676	0.154	-4.390	0.000	-0.978	-0.374
Kaffrine	-0.428	0.187	-2.290	0.022	-0.795	-0.061
Kedougou	-0.360	0.276	-1.300	0.192	-0.901	0.181
Sedhiou	-0.436	0.176	-2.480	0.013	-0.780	-0.092

Facility-based childbirth

Logistic regression number of obs.	=	6559
LR chi2(26)	=	660.110
Prob > chi2	=	0.000
Log likelihood	=	-2152.222
Pseudo R2	=	0.133

**Facility-based childbirth**	Coef.	Std. Err.	z	P>z	95% Conf. interval	
cons	3.177	0.384	8.270	0.000	2.424	3.930
Age	0.044	0.009	4.840	0.000	0.026	0.062
Children ever born	-0.213	0.027	-7.890	0.000	-0.266	-0.160
2^0 / 30 education (ref.)						
No education	-1.016	0.169	-6.030	0.000	-1.347	-0.686
1^0 education	-0.476	0.182	-2.620	0.009	-0.833	-0.120
Urban(ref.)						
rural	-0.905	0.108	-8.350	0.000	-1.117	-0.692
Catholic / protestant (ref.)						
Islam	0.094	0.267	0.350	0.726	-0.429	0.617
Traditional / Other	-0.618	0.699	-0.880	0.376	-1.989	0.752
No partner	-0.108	0.187	-0.580	0.562	-0.475	0.258
No complication information	-0.464	0.087	-5.330	0.000	-0.635	-0.294
Dakar (ref.)						
Ziguinchor	-0.142	0.293	-0.490	0.627	-0.718	0.433
Diourbel	-0.005	0.171	-0.030	0.975	-0.340	0.329
SaintLouis	-0.188	0.205	-0.920	0.359	-0.589	0.214
Tambacounda	-1.075	0.228	-4.710	0.000	-1.522	-0.627
Kaolack	-0.462	0.186	-2.480	0.013	-0.827	-0.097
This	0.496	0.181	2.740	0.006	0.141	0.850
Louga	-0.184	0.199	-0.920	0.356	-0.574	0.207
Fatick	-0.199	0.215	-0.930	0.355	-0.620	0.222
Kolda	-1.561	0.219	-7.130	0.000	-1.990	-1.131
Matam	-0.517	0.214	-2.420	0.015	-0.936	-0.099

Kaffrine	-0.746	0.242	-3.080	0.002	-1.220	-0.272
Kedougou	-1.513	0.312	-4.840	0.000	-2.125	-0.901
Sedhiou	-1.310	0.229	-5.710	0.000	-1.760	-0.861

C-section

Logistic regression number of obs.	=	6479
LR chi2(26)	=	218.250
Prob > chi2	=	0.000
Log likelihood	=	-1589.791
Pseudo R2	=	0.064

**C-section**	Coef.	Std. Err.	z	P>z	95% Conf. interval	
cons	-3.225	0.354	-9.100	0.000	-3.919	-2.530
Age	0.081	0.009	8.600	0.000	0.062	0.099
Children ever born	-0.240	0.035	-6.860	0.000	-0.309	-0.171
2^0 / 30 education (ref.)						
No education	-0.314	0.135	-2.330	0.020	-0.579	-0.050
1^0 education	-0.306	0.136	-2.250	0.025	-0.573	-0.039
Urban(ref.)						
rural	-0.273	0.129	-2.110	0.035	-0.526	-0.020
Catholic / protestant (ref.)						
Islam	-0.340	0.219	-1.550	0.121	-0.769	0.090
Traditional / Other	0.203	0.928	0.220	0.827	-1.617	2.022
No partner	0.452	0.159	2.840	0.004	0.140	0.763
No complication information	-0.512	0.103	-4.960	0.000	-0.715	-0.310
Dakar (ref.)						
Ziguinchor	-0.489	0.274	-1.790	0.074	-1.026	0.048
Diourbel	0.279	0.175	1.590	0.111	-0.064	0.622
SaintLouis	0.110	0.207	0.530	0.596	-0.296	0.515
Tambacounda	-0.537	0.397	-1.350	0.176	-1.315	0.240
Kaolack	-0.543	0.239	-2.270	0.023	-1.011	-0.074
This	-0.384	0.170	-2.270	0.023	-0.717	-0.052
Louga	0.076	0.225	0.340	0.736	-0.365	0.517
Fatick	-0.646	0.315	-2.050	0.040	-1.263	-0.029
Kolda	-0.627	0.385	-1.630	0.104	-1.382	0.129
Matam	-0.282	0.330	-0.860	0.392	-0.928	0.364
Kaffrine	-0.594	0.473	-1.260	0.209	-1.521	0.333
Kedougou	-0.464	0.678	-0.680	0.494	-1.793	0.865
Sedhiou	-0.805	0.416	-1.940	0.053	-1.619	0.010

Supplementary Table 2: Sensitivity analysis by using the coefficients of the upper three quintiles against the lower two quintiles

Burkina Faso

	Family planning exposure	Modern contraceptive	Antenatal	FBD	C-Section
Obs. N	9831	9838	9830	9832	9838
N of Obs G=*lw*	3668	3669	3667	3665	3669
N of Obs G=*hw*	6163	6169	6163	6167	6169
Pr (low-wealth)	0.49	0.09	0.28	0.62	0.01
Pr (high-wealth)	0.64	0.20	0.40	0.84	0.03
Difference	-0.15	-0.12	-0.12	-0.22	-0.02
Total explained	-0.06	-0.07	-0.08	-0.14	-0.02
% explained	42%	60%	70%	62%	94%

Variable contribution

	Family planning exposure	Modern contraceptive	Antenatal	FBD	C-Section
Age	0.00*	0.00	0.00*	-0.00	0.00
	(0.00)	(0.00)	(0.00)	(0.00)	(0.00)
Children ever born	-0.01	0.00	-0.02***	-0.01*	-0.00
	(0.00)	(0.00)	(0.00)	(0.00)	(0.00)
No education	-0.03***	-0.04***	-0.03***	-0.02***	-0.00
	(0.00)	(0.00)	(0.00)	(0.01)	(0.00)
1^0 education	0.01***	0.01***	0.01***	0.01	0.00
	(0.00)	(0.00)	(0.00)	(0.01)	(0.00)
Rural	-0.04***	-0.03***	-0.00	-0.02***	-0.01***
	(0.00)	(0.00)	(0.00)	(0.00)	(0.00)
No religion	-0.00	0.00	-0.00	0.00	0.00
Islam	(0.00)	(0.00)	(0.00)	(0.00)	(.)
Animist	-0.00	0.00***	0.00*	0.01**	0.00
	(0.00)	(0.00)	(0.00)	(0.00)	(0.00)
Traditional / Other	-0.00	-0.01***	-0.01**	-0.02***	-0.00
	(0.00)	(0.00)	(0.00)	(0.00)	(0.00)
No partner	-0.00	0.00***	0.00*	-0.00	0.00
	(0.00)	(0.00)	(0.00)	(0.00)	(0.00)
No Complication information	-0.00	-0.00**	-0.00	-0.01***	0.00
	(0.00)	(0.00)	(0.00)	(0.00)	(0.00)
Health facility permit	-0.00*	-0.00	-0.00**	-0.00**	0.00
	(0.00)	(0.00)	(0.00)	(0.00)	(0.00)
Health facility money	-0.01***	-0.00*	-0.00	-0.00	-0.00
	(0.00)	(0.00)	(0.00)	(0.00)	(0.00)
Health facility distance	-0.01***	-0.00	-0.00*	-0.02***	-0.00
	(0.00)	(0.00)	(0.00)	(0.00)	(0.00)

Health facility alone	-0.00	-0.00	-0.00	-0.00	0.00
	(0.00)	(0.00)	(0.00)	(0.00)	(0.00)
Cascades	-0.01***	-0.00*	0.00	-0.00*	0.00
	(0.00)	(0.00)	(0.00)	(0.00)	(0.00)
Centre	-0.02***	-0.01*	-0.02***	-0.01***	-0.00
	(0.00)	(0.00)	(0.00)	(0.00)	(0.00)
Centre Est	-0.01***	0.00	-0.01***	-0.01***	0.00
	(0.00)	(0.00)	(0.00)	(0.00)	(0.00)
Centre Nord	0.00***	-0.00	-0.00	0.00	-0.00
	(0.00)	(0.00)	(0.00)	(0.00)	(0.00)
Centre Ouest	-0.00*	-0.00	-0.00	-0.00	-0.00
	(0.00)	(0.00)	(0.00)	(0.00)	(0.00)
Centre Sud	-0.00	0.00	-0.00	-0.00***	0.00
	(0.00)	(0.00)	(0.00)	(0.00)	(0.00)
Est	0.03***	0.01*	0.00	-0.01	-0.00
	(0.00)	(0.00)	(0.00)	(0.00)	(0.00)
Hauts Basins	-0.01***	-0.01***	0.00	0.00	0.00
	(0.00)	(0.00)	(0.00)	(0.00)	(0.00)
Nord	-0.00	0.00	-0.00	0.00	-0.00
	(0.00)	(0.00)	(0.00)	(0.00)	(0.00)
Plateau Central	-0.01***	-0.00	-0.00	-0.00*	-0.00
	(0.00)	(0.00)	(0.00)	(0.00)	(0.00)
Sahel	0.03***	0.00	-0.02***	-0.02**	-0.00
	(0.00)	(0.00)	(0.00)	(0.01)	(0.00)
Sud Ouest	0.01***	0.00	0.00	-0.00	-0.00
	(0.00)	(0.00)	(0.00)	(0.00)	(0.00)
N	9831	9838	9830	9832	9838

Standard errors in parentheses

="* p<0.05 ** p<0.01 *** p<0.001"

Note: Standard errors are reported in parentheses below contribution estimates.

Niger

	Family planning exposure	Modern contraceptive	Antenatal	FBD	C-Section
Obs. N	7645	7654	7619	7652	7654
N of obs G=*lw*	2629	2632	2626	2631	2632
N of obs G=*hw*	5016	5022	4993	5021	5022
Pr(low-wealth)	0.36	0.10	0.27	0.19	0.01
Pr(high-wealth)	0.56	0.18	0.36	0.43	0.02
Difference	-0.20	-0.08	-0.09	-0.25	-0.01
Total explained	-0.10	-0.06	-0.03	-0.18	-0.01
% explained	52%	80%	38%	73%	92%
Variable contribution					
	Family planning exposure	Modern contraceptive	Antenatal	FBD	C-Section
Age	0.00**	-0.00	0.00	0.00	0.00
	(0.00)	(0.00)	(0.00)	(0.00)	(0.00)
Children ever born	-0.00	0.00	-0.00*	-0.01***	-0.00
	(0.00)	(0.00)	(0.00)	(0.00)	(0.00)
No education	-0.02***	-0.02***	-0.02***	-0.02***	-0.00*
	(0.00)	(0.00)	(0.00)	(0.00)	(0.00)
1^0 education	0.00*	0.00**	0.01***	0.00	0.00
	(0.00)	(0.00)	(0.00)	(0.00)	(0.00)
Rural	-0.03***	-0.03***	-0.01	-0.07***	-0.00**
	(0.00)	(0.00)	(0.00)	(0.00)	(0.00)
No partner	0.00	0.00***	-0.00	-0.00	-0.00
	(0.00)	(0.00)	(0.00)	(0.00)	(0.00)
No Complication information	-0.01***	-0.00	-0.00	-0.01***	-0.00
	(0.00)	(0.00)	(0.00)	(0.00)	(0.00)
Don't know	-0.00***	0.00	-0.00	-0.00	0.00
	(0.00)	(0.00)	(0.00)	(0.00)	(0.00)
Missing	-0.03***	-0.01***	0.00	-0.03***	-0.00
	(0.00)	(0.00)	(.)	(0.00)	(0.00)
Health facility permit	-0.00**	0.00	-0.00	-0.00*	-0.00
	(0.00)	(0.00)	(0.00)	(0.00)	(0.00)
Health facility money	-0.00	0.00	-0.00	0.00	-0.00
	(0.00)	(0.00)	(0.00)	(0.00)	(0.00)
Health facility distance	-0.01***	-0.00	-0.00*	-0.01***	-0.00
	(0.00)	(0.00)	(0.00)	(0.00)	(0.00)
Health facility alone	0.00***	-0.00	0.00	-0.00	-0.00
	(0.00)	(0.00)	(0.00)	(0.00)	(0.00)
Agadez	-0.00	0.00	-0.00	0.00	0.00
	(0.00)	(0.00)	(0.00)	(0.00)	(0.00)
Diffa	0.00***	0.00	0.00	-0.00	0.00
	(0.00)	(0.00)	(0.00)	(0.00)	(0.00)
Dosso	0.00*	-0.00	0.00*	0.00	0.00
	(0.00)	(0.00)	(0.00)	(0.00)	(0.00)
Maradi	-0.00	-0.00	-0.00	-0.00	-0.00
	(0.00)	(0.00)	(0.00)	(0.00)	(0.00)

Tahoua	-0.00		-0.01**	-0.00	-0.00	-0.00
	(0.00)		(0.00)	(0.00)	(0.00)	(0.00)
Tillaberi	-0.01***		-0.00	0.00**	0.00	-0.00
	(0.00)		(0.00)	(0.00)	(0.00)	(0.00)
Zinder	-0.00		-0.00	0.00	-0.01*	-0.00
	(0.00)		(0.00)	(0.00)	(0.00)	(0.00)

Standard errors in parentheses
="* p<0.05 ** p<0.01 *** p<0.001"

Nigeria

	Family planning exposure	Modern contraceptive	Antenatal	FBD	C-Section
Obs. N	19911	19959	19427	19919	19959
N of obs G=*lw*	8859	8873	8744	8853	8873
N of obs G=*hw*	11052	11086	10683	11066	11086
Pr(low-wealth)	0.13	0.03	0.27	0.12	0.01
Pr(high-wealth)	0.50	0.17	0.75	0.59	0.04
Difference	-0.37	-0.14	-0.48	-0.46	-0.03
Total explained	-0.26	-0.12	-0.16	-0.42	-0.03
% explained	71%	85%	33%	90%	101%
Variable contribution					
	Family planning exposure	Modern contraceptive	Antenatal	FBD	C-Section
Age	-0.00*	0.00	-0.00**	-0.01***	-0.00
	(0.00)	(0.00)	(0.00)	(0.00)	(0.00)
Children ever born	-0.00	0.01***	-0.01***	-0.02***	-0.00**
	(0.00)	(0.00)	(0.00)	(0.00)	(0.00)
No education	-0.12***	-0.05***	-0.04***	-0.08***	-0.01***
	(0.01)	(0.00)	(0.01)	(0.01)	(0.00)
1^0 education	0.01***	0.00	0.01***	0.01***	-0.00
	(0.00)	(0.00)	(0.00)	(0.00)	(0.00)
Rural	-0.05***	-0.01***	-0.01*	-0.04***	-0.00***
	(0.00)	(0.00)	(0.00)	(0.00)	(0.00)
Islam	0.02***	-0.02***	0.00	-0.03***	-0.00**
	(0.00)	(0.00)	(0.00)	(0.00)	(0.00)
Traditional / Other	-0.00	-0.00*	0.00	-0.00	0.00
	(0.00)	(0.00)	(0.00)	(0.00)	(0.00)
No partner	0.00*	-0.00	0.00	0.00**	0.00
	(0.00)	(0.00)	(0.00)	(0.00)	(0.00)
No Complication information	0.00***	-0.00	0.00***	-0.00	-0.00*
	(0.00)	(0.00)	(0.00)	(0.00)	(0.00)
Don't know	-0.00**	-0.00*	0.00	-0.00**	0.00
	(0.00)	(0.00)	(0.00)	(0.00)	(0.00)
Missing	-0.08***	-0.02***	0.00	-0.13***	-0.01***
	(0.00)	(0.00)	(.)	(0.00)	(0.00)
Health facility permit	-0.00	-0.00**	-0.00	-0.00	0.00
	(0.00)	(0.00)	(0.00)	(0.00)	(0.00)
Health facility money	-0.00***	0.00	-0.00**	-0.00***	-0.00*
	(0.00)	(0.00)	(0.00)	(0.00)	(0.00)
Health facility distance	-0.00	-0.00	-0.02***	-0.01***	-0.00
	(0.00)	(0.00)	(0.00)	(0.00)	(0.00)
Health facility alone	0.00	-0.00	0.00	-0.00	-0.00
	(0.00)	(0.00)	(0.00)	(0.00)	(0.00)
Health facility attitude	0.01***	0.00	0.00	0.00**	0.00
	(0.00)	(0.00)	(0.00)	(0.00)	(0.00)

North Central	0.02***	0.00	0.03***	0.00	-0.00
	(0.00)	(0.00)	(0.00)	(0.00)	(0.00)
North East	-0.03***	-0.01***	-0.04***	-0.03***	0.00
	(0.00)	(0.00)	(0.00)	(0.00)	(0.00)
North West	-0.04***	-0.02***	-0.07***	-0.06***	-0.00
	(0.00)	(0.00)	(0.01)	(0.00)	(0.00)
South East	0.01***	0.00***	0.01*	-0.00***	-0.00
	(0.00)	(0.00)	(0.00)	(0.00)	(0.00)
South South	0.02***	0.00***	0.02***	0.01***	-0.00
	(0.00)	(0.00)	(0.00)	(0.00)	(0.00)

Standard errors in parentheses

="* p<0.05 ** p<0.01 *** p<0.001"

Ghana

	Family planning exposure	Modern contraceptive	Antenatal	FBD	C-Section
Obs. N	4.147	4.147	4.127	4.146	4.147
N of obs G=*lw*	2125	2125	2111	2125	2125
N of obs G=*hw*	2022	2022	2016	2021	2022
Pr(low-wealth)	0.5219482	0.2531611	0.8403013	0.5746376	0.062175
Pr(high-wealth)	0.7805742	0.2575787	0.9422785	0.8988781	0.1909415
Difference	-0.258626	-0.0044176	-0.1019772	-0.3242406	-0.1287665
Total explained	-0.1324507	0.0692372	-0.0932167	-0.2083955	-0.0994106
% explained	51%	-1567%	91%	64%	77%

Variable contribution

	Family planning exposure	Modern contraceptive	Antenatal	FBD	C-Section
Age	0.00	-0.00	0.01	0.01*	0.00
	(0.00)	(0.00)	(0.01)	(0.00)	(0.01)
Children ever born	-0.01	0.05***	-0.02*	-0.06***	-0.06***
	(0.01)	(0.01)	(0.01)	(0.01)	(0.01)
No education	-0.05***	-0.02	-0.03*	-0.03**	-0.01
	(0.01)	(0.01)	(0.01)	(0.01)	(0.01)
1^0 education	-0.01**	-0.00	-0.00	-0.00	-0.00
	(0.00)	(0.00)	(0.00)	(0.00)	(0.00)
Rural	0.02	0.01	-0.01	-0.07***	-0.01
	(0.01)	(0.01)	(0.01)	(0.01)	(0.01)
Islam	0.00	-0.00	0.00	0.00	0.00
	(0.00)	(0.00)	(0.00)	(0.00)	(0.00)
Traditional / Other	0.01	0.00	-0.00	0.00	0.01
	(0.00)	(0.01)	(0.00)	(0.01)	(0.00)
No partner	0.00	-0.00	0.00	0.00	-0.00
	(0.00)	(0.00)	(0.00)	(0.00)	(0.00)
No Complication information	-0.01*	-0.00	-0.00*	-0.00*	-0.00*
	(0.00)	(0.00)	(0.00)	(0.00)	(0.00)
Health facility permit	0.00	0.00	0.00	-0.00	-0.00
	(0.00)	(0.00)	(0.00)	(0.00)	(0.00)
Health facility money	-0.01*	-0.00	-0.01	0.00	-0.00
	(0.00)	(0.01)	(0.00)	(0.01)	(0.00)
Health facility distance	-0.00	-0.01	-0.00	-0.00	0.01
	(0.01)	(0.01)	(0.01)	(0.01)	(0.00)
Health facility alone	-0.00	0.00	-0.01	-0.01	0.00
	(0.00)	(0.00)	(0.00)	(0.00)	(0.00)
No insurance	-0.00	0.00	-0.00	-0.00	-0.00
	(0.00)	(0.00)	(0.00)	(0.00)	(0.00)
Western	-0.01***	-0.00*	0.00	0.01*	0.00
	(0.00)	(0.00)	(0.00)	(0.00)	(0.00)

Central	-0.01***	-0.00***	0.01	0.01**	-0.00
	(0.00)	(0.00)	(0.00)	(0.00)	(0.00)
Volta	0.00	0.00*	-0.00	-0.00	-0.00**
	(0.00)	(0.00)	(0.00)	(0.00)	(0.00)
Eastern	-0.00	0.00	-0.00	0.00	-0.00
	(0.00)	(0.00)	(0.00)	(0.00)	(0.00)
Greater Accra	-0.02***	-0.01	0.03**	-0.00	0.01
	(0.01)	(0.01)	(0.01)	(0.01)	(0.00)
Brong Ahafo	-0.01***	0.01**	-0.00	0.00	-0.00*
	(0.00)	(0.00)	(0.00)	(0.00)	(0.00)
Northern	-0.01	0.00	-0.05*	-0.06**	-0.02*
	(0.02)	(0.01)	(0.02)	(0.02)	(0.01)
Upper East	-0.02**	0.02**	-0.00	0.00	-0.01
	(0.01)	(0.01)	(0.01)	(0.01)	(0.00)
Upper West	0.01	0.01	-0.00	0.00	-0.00
	(0.00)	(0.01)	(0.00)	(0.00)	(0.00)
N	4147	4147	4127	4146	4147

Standard errors in parentheses
="* p<0.05 ** p<0.01 *** p<0.001"
Note: Standard errors are reported in parantheses below contribution estimates.

Senegal

	Family planning exposure	Modern contraceptive	Antenatal	FBD	C-Section
Obs. N	8.839	8.839	8.690	8.839	8.750
N of obs G=*lw*	4932	4932	4872	4932	4877
N of obs G=*hw*	3907	3907	3818	3907	3873
Pr(low-wealth)	0.3326594	0.1753225	0.4047448	0.6155451	0.0328351
Pr(high-wealth)	0.6070043	0.3396693	0.6257858	0.9276707	0.0855921
Difference	-0.274345	-0.1643468	-0.2210409	-0.3121256	-0.052757
Total explained	-0.0927315	-0.0865574	-0.1016396	-0.1004877	-0.0386613
% explained	34%	53%	46%	32%	73%

Variable contribution

	Family planning exposure	Modern contraceptive	Antenatal	FBD	C-Section
Age	-0.00*	0.00	-0.01***	-0.00	-0.01*
	(0.00)	(0.00)	(0.00)	(0.00)	(0.00)
Children ever born	-0.00	0.03***	-0.04***	-0.02***	-0.01***
	(0.00)	(0.00)	(0.00)	(0.01)	(0.00)
No education	-0.05***	-0.04***	-0.04***	-0.02***	-0.01**
	(0.01)	(0.01)	(0.01)	(0.00)	(0.00)
1^0 education	0.01**	0.00	0.01***	0.00	0.00*
	(0.00)	(0.00)	(0.00)	(0.00)	(0.00)
Rural	-0.01	-0.05***	-0.03**	-0.03***	-0.01
	(0.01)	(0.01)	(0.01)	(0.01)	(0.01)
Islam	0.00	0.00*	0.00	0.00	0.00
	(0.00)	(0.00)	(0.00)	(0.00)	(0.00)
Animist	-0.00**	0.00	-0.00	-0.00*	0.00
	(0.00)	(0.00)	(0.00)	(0.00)	(0.00)
No partner	0.00**	0.00***	0.00***	-0.00	-0.00*
	(0.00)	(0.00)	(0.00)	(0.00)	(0.00)
No Complication infomation	0.00**	0.00	0.01***	0.00	0.00**
	(0.00)	(0.00)	(0.00)	(0.00)	(0.00)
Ziguinchor	0.00	-0.00	0.00	-0.00	-0.00
	(0.00)	(0.00)	(0.00)	(0.00)	(0.00)
Diourbel	0.01***	0.01***	0.00*	-0.00	-0.00
	(0.00)	(0.00)	(0.00)	(0.00)	(0.00)
SaintLouis	-0.00**	0.00	-0.00	-0.00	0.00
	(0.00)	(0.00)	(0.00)	(0.00)	(0.00)
Tambacounda	-0.01	-0.01*	0.00	-0.01*	-0.00
	(0.00)	(0.00)	(0.00)	(0.00)	(0.00)
Kaolack	-0.00	-0.00*	0.00	-0.00	-0.00**
	(0.00)	(0.00)	(0.00)	(0.00)	(0.00)
This	0.00	0.00	0.00	-0.00*	0.00*
	(0.00)	(0.00)	(0.00)	(0.00)	(0.00)

Louga	-0.01***	-0.00	-0.00*	0.00	0.00
	(0.00)	(0.00)	(0.00)	(0.00)	(0.00)
Fatick	-0.01**	-0.00	-0.00	-0.00	-0.00
	(0.00)	(0.00)	(0.00)	(0.00)	(0.00)
Kolda	-0.02**	-0.01***	-0.01	-0.02**	-0.00
	(0.01)	(0.00)	(0.01)	(0.01)	(0.00)
Matam	-0.01***	-0.00***	-0.00	0.00	-0.00
	(0.00)	(0.00)	(0.00)	(0.00)	(0.00)
Kaffrine	-0.00	-0.01*	0.00	-0.00	-0.00
	(0.01)	(0.00)	(0.01)	(0.01)	(0.00)
Kedougou	-0.00	-0.00	0.00	-0.00	0.00
	(0.00)	(0.00)	(0.00)	(0.00)	(0.00)
Sedhiou	-0.01*	-0.01**	-0.00	-0.01*	-0.00
	(0.00)	(0.00)	(0.00)	(0.00)	(0.00)
N	8839	8839	8690	8839	8750

Standard errors in parentheses
="* p<0.05 ** p<0.01 *** p<0.001"
Note: Standard errors are reported in parantheses below contribution estimates.

Supplementary Table 3: Summary of regional covariates included in the analysis

Country / Region	low-wealth		higher wealth	
**Burkina Faso**	mean	sd	mean	sd
Boucle~n	0.07	0.26	0.12	0.33
Cascades	0.01	0.10	0.05	0.21
Centre	0.01	0.11	0.12	0.32
Centre Est	0.05	0.22	0.09	0.28
Centre Nord	0.08	0.27	0.08	0.27
Centre Ouest	0.08	0.27	0.08	0.27
Centre Sud	0.04	0.20	0.05	0.22
Est	0.22	0.41	0.08	0.27
Hauts Basins	0.06	0.23	0.12	0.33
Nord	0.06	0.23	0.08	0.27
Plateau Central	0.03	0.16	0.05	0.22
Sahel	0.20	0.40	0.06	0.23
Sud Ouest	0.09	0.29	0.03	0.17
**Niger**				
Agadez	0.02	0.14	0.01	0.12
Diffa	0.02	0.14	0.03	0.17
Dosso	0.09	0.29	0.13	0.34
Maradi	0.21	0.41	0.22	0.41
Tahoua	0.28	0.45	0.20	0.40
Tillaberi	0.12	0.32	0.13	0.34
Zinder	0.25	0.44	0.19	0.40
Niamey	0.00	0.00	0.07	0.26
**Nigeria**				
North Central	0.07	0.26	0.16	0.37
North East	0.30	0.46	0.13	0.34
North West	0.60	0.49	0.29	0.46
South East	0.02	0.15	0.10	0.30
South South	0.00	0.05	0.13	0.33
South West	0.01	0.11	0.19	0.39
**Ghana**				
Western	0.03	0.16	0.12	0.33
Central	0.02	0.13	0.13	0.34
Volta	0.02	0.12	0.20	0.40
Eastern	0.08	0.27	0.07	0.26
Greater Accra	0.06	0.24	0.10	0.30
Ashanti	0.05	0.21	0.21	0.41
Brong Ahafo	0.10	0.31	0.09	0.28
Northern	0.41	0.49	0.04	0.19
Upper East	0.16	0.36	0.01	0.11
Upper West	0.08	0.27	0.01	0.11
**Senegal**				
Dakar	0.00	0.00	0.26	0.44
Ziguinchor	0.01	0.10	0.04	0.19

Diourbel	0.07	0.25	0.15	0.36
SaintLouis	0.06	0.24	0.06	0.24
Tambacounda	0.15	0.35	0.03	0.16
Kaolack	0.11	0.31	0.08	0.27
This	0.03	0.17	0.15	0.36
Louga	0.11	0.31	0.07	0.25
Fatick	0.07	0.25	0.05	0.21
Kolda	0.15	0.36	0.03	0.17
Matam	0.04	0.19	0.04	0.19
Kaffrine	0.14	0.35	0.02	0.15
Kedougou	0.02	0.14	0.01	0.10
Sedhiou	0.05	0.22	0.03	0.16

Supplementary B: Fairlie decomposition detailed

The average difference in use of a reproductive health care service between the wealth groups can be expressed as:

$$\bar{Y}^{lw} - \bar{Y}^{hw} = \left[\sum_{i=1}^{N^{lw}} \frac{F(X_i^{lw}\widehat{\beta}^{lw})}{N^{lw}} - \sum_{i=1}^{N^{hw}} \frac{F(X_i^{hw}\widehat{\beta}^{lw})}{N^{hw}}\right] + \left[\sum_{i=1}^{N^{hw}} \frac{F(X_i^{hw}\widehat{\beta}^{lw})}{N^{hw}} - \sum_{i=1}^{N^{hw}} \frac{F(X_i^{hw}\widehat{\beta}^{hw})}{N^{hw}}\right] \quad (1)$$

where $\overline{Y}$ is the average probability of use of a reproductive health care service by a group (a dummy variable which, depending on the analysis, includes whether a woman had at least one family planning information source, used modern contraceptives, 4+ antenatal care visits, facility-based childbirth, C-section respectively), *lw* indicates the low-wealth group and *hw* indicates the high-wealth group. X_i is the vector of independent variables of respondent *i* in a given group (low-wealth or high-wealth group), $\hat{\beta}$ is the vector of coefficients estimated separately for each of the two groups using the pooled sample, *N* is the number of observations in a given group, and *F* is the cumulative logistic distribution function.

The first term on the right-hand side of equation (*1*) measures the proportion of the wealth gap in the use of a selected reproductive health care service that is due to differences in the distribution of characteristics of the two groups. This is the variation explained by differences in observable characteristics. This represents the extent to which the wealth gap in reproductive health care services would reduce if the low-wealth group had the same characteristics as the high-wealth group. The second term captures the degree to which women in the low-wealth and high-wealth groups, with similar observable characteristics, have different propensities in use. This indicates the portion of the gap that is due to differences in the impact of observable characteristics between the low- and high-wealth groups. This is the portion of the gap that may be due to wealth discrimination, differences in the availability of health care services, differences in attitudes between wealth groups, or other unmeasured characteristics (Fairlie, 2006).
